# Prognostic gene expression signature revealed the involvement of mutational pathways in cancer genome

**DOI:** 10.7150/jca.40237

**Published:** 2020-05-18

**Authors:** Hongde Liu, Huamei Li, Kun Luo, Amit Sharma, Xiao Sun

**Affiliations:** 1 State Key Laboratory of Bioelectronics, School of Biological Science & Medical Engineering, Southeast University, Nanjing 210096, China; 2 Department of Neurosurgery, Xinjiang Evidence-Based Medicine Research Institute, First Affiliated Hospital of Xinjiang Medical University, Urumqi 830054, China; 3 Department of Ophthalmology, University Hospital Bonn, Germany.

**Keywords:** Prognostic gene, Cancer, Mutation, Diagnosis

## Abstract

**Background**: Over the years, many efforts have been made to use the gene expression profiles of cancer types/subtypes to identify the prognostic genes with their potential clinical applications. However, one major challenge remains is to predict the common prognostic genes using simultaneously the dataset of multiple cancers, especially by considering the differences in survival, expression and the associated mutated pathways.

**Methods**: Herein, we carried out a comprehensive examination for the prognostic genes and linked them to the mutational status of 29 cancers, so as to find independent prognostic genes and mechanisms. Additionally, their diagnostic value of them was also assessed. **Results**: our extensive analysis revealed: 1) the number of prognostic and diagnostic genes differs greatly across the cancers, 2) the potentially implicated 22 genes harbor the diagnostic as well as prognostic capacity, 3) the universal prognostic genes (*CDC20*, *CDCA8*, *ASPM*, *ERCC6L*, and *GTSE1*) were found to be involved in the spindle assembly checkpoint, 4) the prognostic genes were found to be statistically linked to the frequently mutated TP53-, MAPK-, PI3K- and AKT- related pathways. We also manually mined possible biological mechanisms for some of the statistical links in literatures.

**Conclusions**: Taken together, we identified the prognostic genes and in addition we assessed their diagnostic capacity. Our analysis provides an important insight about the considerable overlapping between gene expression variation and the further associated altered mutational pathways across the cancer genome. We thus hypothesized that cancer related (mutated) genes are tightly connected and are capable to reshape the genome in multiple cancer types.

## Introduction

Cancers are characterized by distinct patterns of mutation and gene expression, associated with different prognosis. Identification of cancer related genes and their relevant pathways is an important step to elucidate the associated molecular mechanisms and biological processes.

Recently, one study summarized that the shorter survival is associated with the upregulation of genes related to cell growth and the downregulation of genes related to cellular differentiation 1. Likewise, the 70-gene signature test (MammaPrint) provided valuable information for considering those breast cancer patients which might benefit from adjuvant chemotherapy 2. Considering metastasis a key determinant of patient survival, several studies also performed similar gene expression evaluations towards accurate prognosis prediction and subsequently proposed this method for therapeutic decisions in the cancers 3-5. Gene expression signature was also found to be useful in predicting the survival stage I in non-small cell lung carcinoma (NSCLC) patients 6. Wilson and colleagues using gene expression signatures identified novel groups of acute myeloid leukemia (AML) which were not predicted before by the traditional studies that impact the prognosis and potential therapy 7. Since, the tumor microenvironment strongly influences cancer development, progression and metastasis, a study took advantage of this biological condition and revealed the prognostic gene-expression signature in NSCLC 8.

Apart from global gene expression, its correlation with the cancer associated/driver gene mutations always gained attention. Recently, 299 driver mutation genes were revealed 9. The study confirmed that microsatellite instability was associated with an improved response to immune checkpoint therapy 9.

However, the previous studies lack the through information about the extent of expression variation in cancer and the diagnostic capacity of the prognostic genes, which might be an important contributor for their clinical applicability. Additionally, the relative contribution of cancer associated mutations on the gene expression levels which could enhance the prognostic spectrum is also not fully exploited yet.

Herein, we aim to identify the distinct and recurrent prognostic genes in 29 cancer types simultaneously mainly considering the differences in survival and the expression in cancers. In addition, we thoroughly investigated the impact of cancer specific mutations on the expression level in individual cancer types. Considering clinical utility, we also highlighted about the genes with both prognosis and diagnosis capacity.

## Materials and Methods

### Datasets

Gene expression data, survival data, and mutation data were retrieved from TCGA project from the initial release of Genomic Data Commons (GDC) in October 2016 using RTCGAToolbox 10. A total of 9523 samples across 29 tumor types were downloaded, including 8811 tumor tissues and 712 non-tumor tissues. The abbreviation for cancer type is in supplementary information. We are interested in overall survival of the patients and hoped to find some common prognostic genes, so we did not perform a further categorization for the patients. All samples are used in survival analysis. Microarray-based gene expression data (gene expression omnibus ID: GSE21501) for pancreatic cancer 11) were retrieved for validating.

### Identification of the prognostic genes

The prognostic genes were identified with a log-rank test in a Kaplan-Meier survival model. In each cancer type, for each gene, patients were classified into two groups, the high-expression group (H) and the low-expression group (L), using the expression median of the gene as a cutoff. In addition, we considered both survival difference (P[SV]) and the expression fold change (FC(H/L)) between the two groups. The area under the curve (AUC) of a receiver operating characteristic (ROC) curve and the expression fold change between the cancer (C) and normal (N) tissues (FC(C/N)) were employed to indicate the diagnosis ability.

### Regression for the expression of the prognostic genes with the mutation counts

The 40 prognostic genes, which were identified with P[SV] ≤ 10^-6^ and FC[H/L] ≥ 4, and the top 200 frequently mutated genes, were included in this section. Firstly, for each prognostic gene, the dependence between its expression and the mutation counts of the 200 mutated genes were tested with a Chi-squared (χ^2^) test for each cancer type. The mutated genes with p-value ≤ 0.001 (χ^2^ test) were included in an enrichment analysis. A cutoff of p-value ≤ 0.05 was used to find the enriched gene ontology (GO) terms and pathways. Upon satisfaction of those criteria, a link between the prognostic gene and the mutated pathway (terms) was counted. This was done for all cancers to see how many cancer types shared the link. Secondly, we carried out a generalized linear regression of the expression of the prognostic gene with the mutation counts of the top 200 frequently mutated genes for each cancer type. The regression generated a set of parameters indicating the contribution of the mutation in explaining the expression level of the prognostic gene. Only mutated genes with a significant parameter were used to construct the network. More details are in Supplementary file.

## Results

### Prognostically relevant genes differ substantially across cancers

**Figure 1:**
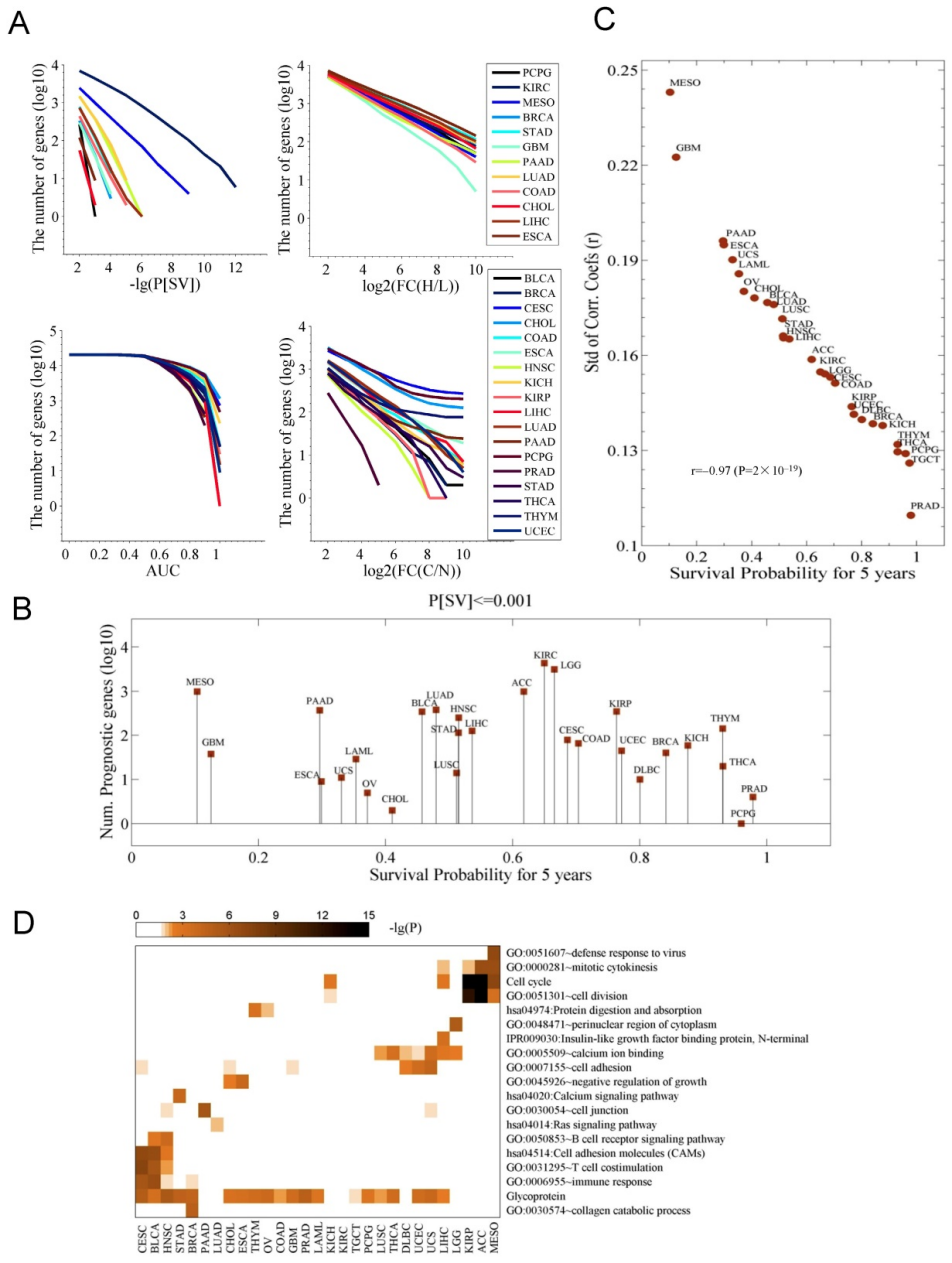
Survival-associated genes differ among cancers. **A**. The number of genes associated with a differential survival (P[SV]), with an expression change in the cancer tissues (FC(H/L)), with a capacity of diagnosis for cancer (AUC), and with an expression change between the control (N) and cancer (C) samples (FC(C/N)), respectively. In each cancer type, for each gene, cancer tissues are divided into high (H) and low (L) expression groups based on the median expression of the gene in the cancer type. P[SV] and FC(H/L) represent survival difference and fold change between the two groups, respectively. FC(C/N) means the fold change between the cancer tissues and normal controls. AUC is the area under receiver operating characteristic (ROC) curves in diagnosing the cancer samples with expression of the gene. **B**. The number of prognostic genes with a significance of P[SV]≤0.001. The data were sorted with the five-year survival probability. **C**. Relationship between survival probability and variation of gene expression among the population. Shown is the five-year survival probability against the standard deviation (Std) of the correlation coefficients of expression profile (20,531 genes) of each pair of patients for each cancer. **D**. The pathways and terms enriched for the genes whose expression was highly correlated to survival time. For each cancer, the top 200 most strongly correlated genes were chosen.

All the identified prognostic genes in this study appeared to significantly differ in all cancer types when checked under the same cutoff of overall survival difference (P[SV]) (Fig. 1A). Some cancer types (CHOL, ESCA, STAD, COAD, GBM, and PCPG) showed a limited number of prognostic genes, while in some cancers (KIRC and MESO), more of the prognostic genes can be identified (Fig. 1A and Fig. S1A). While comparing cancer and control data, numbers of the diagnostic genes were also found to be significantly different in cancers (AUC≥0.9) (the bottom left panel in Fig. 1A, Fig. S1A). In the comparisons for number of the genes with an altered expression between cancer tissues (FC(H/L)) or in comparison to controls (FC(C/N) revealed the quite similar results (two left panels in Fig. 1A), hence, indicating that number of prognostic and diagnostic genes differs in and among cancers.

We did not observe any link between the number of prognostic genes and the five-year overall survival (Fig. 1B). However, the Pearson correlation coefficients (PCCs) of the gene expression of 25,301 genes for each pair of patients for individual cancer, and the associated five-year survival probability revealed that the survival probability is highly correlated to the standard deviation (Std) of the PCC (r=-0.97) (Fig. 1C). On clinical grounds this data requires further validations. The pathways and GO terms further discriminated the cancer types and associated survival-related genes and showed enrichment for cell cycle (in KIRP, ACC, and MESO), T cell costimulation (in CESC, BRCA, STAD, BLCA, HNSC) and cell adhesion (in LUSC, THCA, DBLC, UCEC, USC and LIHC) (Fig. 1D). GBM, LAML, and PAAD were not included in any of the clusters mentioned above. To note, we also observed a substantial enrichment in the term “Glycoprotein” in this analysis. Previously, one study showed the involvement of P-glycoprotein in the multidrug resistance (MDR) phenotype of adult solid tumors 12. The activation status of pathway was also determined for the genes whose expressions change greatly in cancer patients is shown in Fig. S1B. We also noticed the genes enriched for Renin secretion in nine cancer types (Fig. S1B).

### Mapping and characterization of Prognostic genes

To identify prognostic genes, we first considered the association between the variation of clinical outcome (here, overall survival) and the gene expression level between the high- and low- expression groups). The survival difference between the two groups was calculated (P[SV]) with Kaplan-Meier survival model. Second, we estimated the ability of a gene/mRNA level to discriminate between the two groups [high (H) and low (L)], namely the difference extent of the altered expression among the two groups. In preliminary investigations with relaxed criteria of P[SV]≤10^-3^ and FC(H/L)≥2, selecting the top 10 genes for each cancer type, we identified 236 genes in 29 cancer types (see Fig. S2A). In Table S1, we listed the genes and their survival difference (P[SV]), expression fold change (FC(H/L)), and the corresponding cancer types. Eight of the 236 genes were previously known in 17 cancers 1 (Fig. S2B). The remaining novel prognostic genes include *C1orf88* (for ACC), *BCL2L14* (BLCA), *TMEM65* (BRCA), *RBM38* (CESC), *ATP13A3* (CHOL), *ATOH1* (CORD), *ATP1A3* (DLBC, UCS), *GRPEL2* (ESCA), *RARRES2* (GBM), *CHGB* (HNSC), *CLDN3* (KICH), *ATP6V1C2* (KIRH), *HOXD10* (KIRP), *TREML2* (LAML), *ISL2* (LGG), *CDC20* (LIHC), *GTSE1* (LUAD), *PAPPA* (LUSC), *CEP55* (MESO), *DYDC2* (OV), *MYEOV* (PAAD), *KIAA0319* (PRAD), *LBH* (STAD), *CILP* (THCA), *PRKCB* (THYM), and *TP53TG3B* (UCEC) (Table S1). Very few genes were found to be shared across cancer types, which were consistent with the literatures 1, 13. It is notable that the subunits of P- and V-ATPases (such as *ATP13A3*, *ATP1A3*, and *ATP6V1C2*) were in the list. These genes are responsible for transporting cations across membranes and organelle acidification 14.

**Figure 2:**
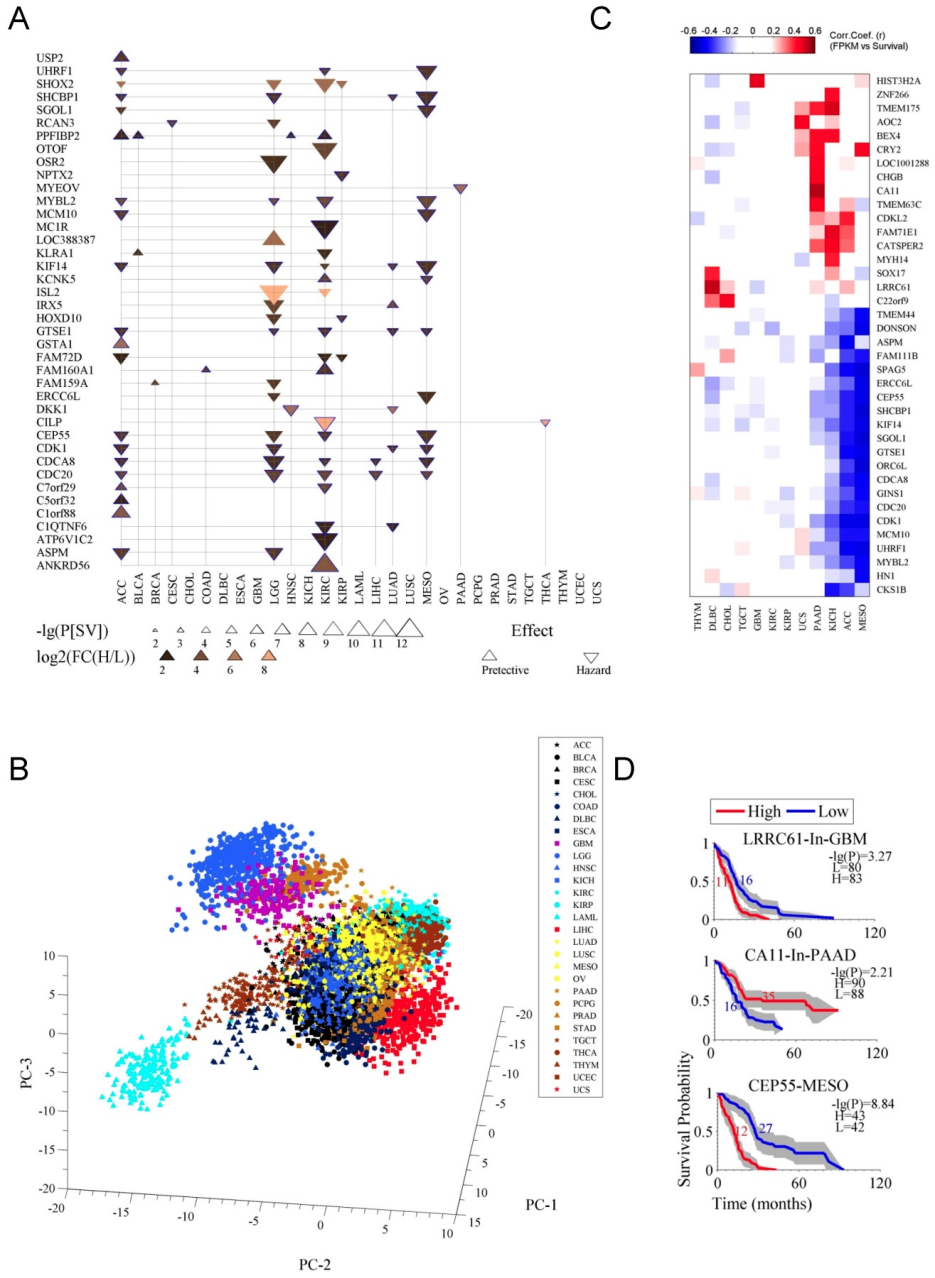
Prognostic genes associated with both survival difference and expression changes in cancer. **A**. The 40 prognostic genes identified. Firstly, with criteria of a survival difference P[SV]≤10^-3^ and fold change of expression FC(H/L)≥2, and by selecting the top ten genes at most for each cancer type, 236 genes were identified in 29 cancer types (see Table S1). Then the 236 genes were further filtered with stricter criteria of P[SV]≤10^-6^ and FC(H/L)≥4. The term “effect” indicates the relationship between gene expression and survival, a downward-pointing triangle means a high expression of the gene corresponds to a poor survival, and an upward-pointing triangle means a high expression to a good survival. **B**. Principal component analysis (PCA) for the gene expression of the 40 prognostic genes (Table S2) in 29 cancer types. **C**. The genes whose expressions highly correlate with survival. The correlation is calculated as a Pearson correlation coefficient (r). **D**. Survival curves for the three prognostic genes.

In second step, we further filtered the prognostic genes by using stricter criteria, P[SV]≤10^-6^ and FC(H/L)≥4, which results into a list of 40 genes (Fig. 2A). The detailed data for the 40 genes was listed in Table S2. Apparently, the cancers like CHOL, ESCA, TGCT, OV and UCS showed no prognostic genes under these criteria (Fig. 2A). The genes *CDC20*, *CDCA8,* and *CEP55* were prognostic in more than three (multiple) cancer types while other genes were specific for particular cancer types, such as *MYEOV* for PAAD (Fig. 2A). Most of the genes had a hazard effect, meaning that high expression of the gene was associated with poorer overall survival (Fig. 2A). The prognostic genes were enriched for the terms of cell cycle, cell division, and cytoskeleton (Fig. S2 C and D). Fig. S2C shows the sub-cellular location of the proteins encoded by the 40 genes. Fig. S2D is for an enrichment analysis for the prognostic genes. Moreover, principal components (PCs) showed clustering patterns for cancer tissues and cancer types (Fig. 2B), indicating that these genes represent cancer-type specific survival information.

As previously mentioned also, that some cancer types had few prognostic genes (Fig. 2A), alternative strategy, using the Pearson correlation coefficient between survival time and gene expression, was applied to identify optional candidates from the 236 prognostic genes (Fig. 2C). Although some of these genes are not in the list in Fig. 2A (see also Table S2), they performed well in indicating survival, for instance, *LRRC61* for GBM, *CA11* for PAAD, and *CEP55* for MESO (Fig. 2C and D). To replicate the previous findings, we also test those known genes in our stringent criteria and found the similar results (Fig. 2D). Specifically, the lower expression of *MYBL2* was associated with a favorable overall survival in LGG 15, LIHC 16 and NSCLC 17, high *DKK1* was identified for gastric cancer 18, and *NPTX2,* which was moderately significant (p ≤ 0.05) for the survival 20, has been suggested to have prognostic value for GBM 19. We also randomly selected *MYEOV*, and tested its prognostic performance with microarray-based data for PAAD 11 and got similar results (Fig. S2E).

Briefly, we systematically identified the independent prognostic genes for each cancer type by considering both survival difference and gene expression discrimination between high- and low- expression patients.

### Genes with both prognostic and diagnostic capacities

**Figure 3:**
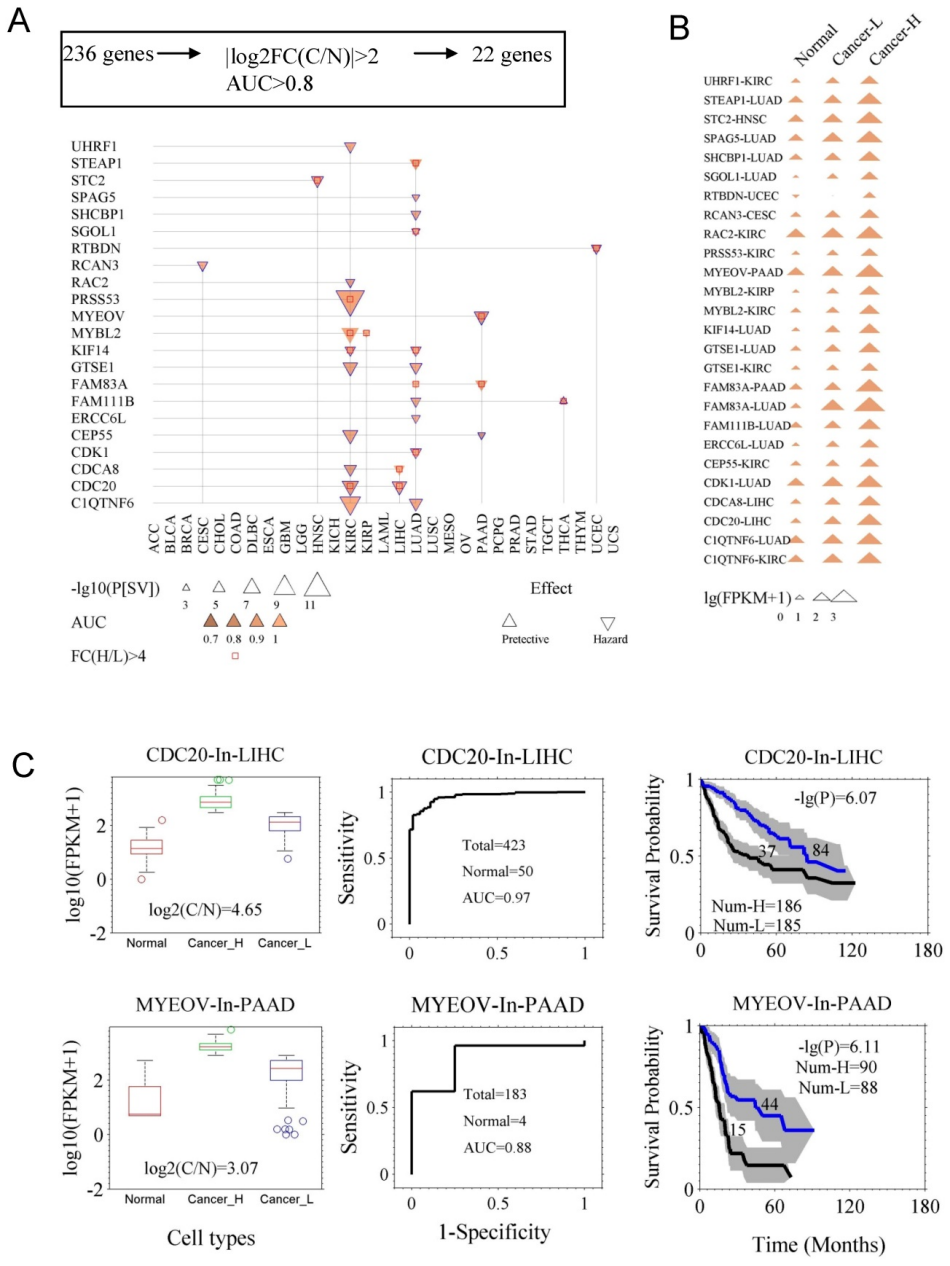
Genes with the capacity for both prognosis and diagnosis. **A**. The 22 genes (Table S3) that are both prognostic and diagnostic. The 236 prognostic genes were further filtered with two new criteria. One was the fold change of expression between the cancer tissues (C) and the normal tissues (N), namely |log_2_(FC[C/N])|≥2. The other was the capacity of differentiating cancer and normal cases, which was assessed by AUC of ROC curve, with the criterion of AUC≥0.8. **B.** The gene expression of the 22 genes in normal tissues, and both low- and high-expression cancer groups (Cancer-L and Cancer-H). **C**. Demonstration of two genes, *CDC20* and *MYEOV*, in prognosis and diagnosis. Left panels, expression levels of the genes in normal and cancer tissues (Cancer-H and Cancer-L). Middle panels, the ROC curves of diagnosis. Right panels, the survival curves of the high- and low-expression groups. The p value of the log-rank test and the number of the groups are indicated.

In case of few genes with prognostic abilities, the average expression levels were higher in normal tissue as compared to the cancer tissues, which could cause suboptimal results while evaluating these genes in clinical application. For example, *DKK1* is prognostic for cancer LUAD, but its expression level even is higher in control (health sample) than in some of LUAD samples (Fig. S3). Therefore, we assessed the diagnostic ability of the 236 prognostic genes from two aspects: the fold change of gene expression between the cancer (C) and the control (N) tissues (FC(C/N)), and the AUC in diagnosis. Using the criteria |log_2_FC(C/N)| ≥ 1.5 and AUC ≥ 0.8, we identified 22 genes (Fig. 3A and Fig. S4). Fig. 3A indicates survival difference P[SV] and diagnostic ability AUC for the 22 genes. Survival curves and ROC curves of the genes are shown in Fig. S4. In Table S3, the detailed AUC and P[SV] are listed for the 22 genes. Here, we also reconfirmed their diagnostic value for *CDC20*, *CDCA8*, *CDK1*, *MYBL2*, *KIF14*, *SPAG5*, *MYEOV*, and *STC2,* while their prognostic value was already identified previously. As shown in Fig. 3B, the expression levels of the genes exhibited a successive increase from the controls, to the low-expression cancer group, and then to the high-expression cancer group, made assessment of both diagnosis and prognosis possible and reliable. In Fig. 3C, we showed capacities of both diagnostic and prognostic for CDC20 and MYEOV. Interestingly, the genes *CDC20*, *CDCA8*, *MYBL2*, *C1QTNF6*, *CEP55*, *CDK1* and *KIF14* were found to be more universal/common, since they mark multiple types of cancers not only in prognosis but also in diagnosis. We propose *CDC20* as a prognostic marker for LIHC and KIRC, and a diagnostic marker for more than nine types of cancer (Fig. S5). The functional aspect linked these genes includes, [*CDC20* (anaphase promoting complex/cyclosome) 21,* CDCA8* (chromosomal passenger complex) 22,* CDK1* and *MYBL2 (*cell cycle progression) 23, 24, *MYBL2* (well-known prognostic predictor) 23 and *KIF14* (chromosome segregation and mitotic spindle formation) 25, 26], to major biological processes.

**Figure 4:**
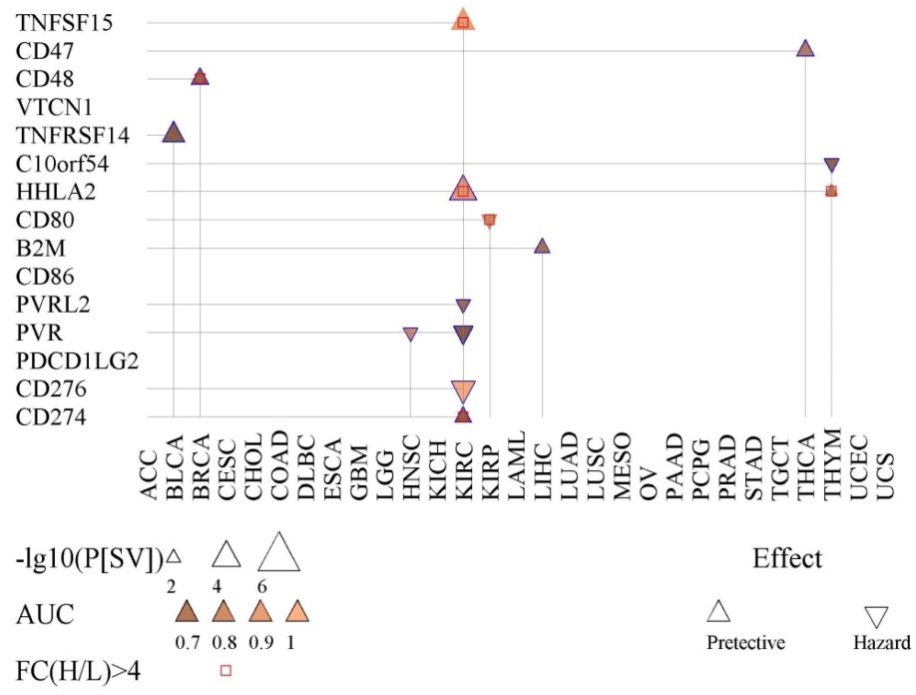
Prognostic and diagnostic value of fifteen immunoregulation-related genes. Shown is the survival difference (P[SV]) between the high- and low-expression groups of each immunoregulation-related gene. The AUC indicates the diagnostic capacity in differentiating cancer tissues.

We also tested the possibility of fifteen immunoregulation-related genes in prognosis and diagnosis (Fig. 4). *PVR* was found to be significant for prognosis for KIRC and HNSC. The product of *PVR* is the ligand of TIGIT, which can repress the activity of NK cells 27. Also, *CD48* showed prognostic value for BRCA (Fig. 4).

### Mutational landscape in cancers and potential association with prognostic genes

To determine the potential role of mutation/s on the expression levels of the 40 prognostic genes with strong criteria (gene list in Table S2), we selected top 200 frequently mutated genes in this analysis (see flowchart in Fig. S6). Fig. S7A shows mutation rate of top 50 mutated genes. Fig. S7B shows an enrichment analysis for the 200 genes.

**Figure 5:**
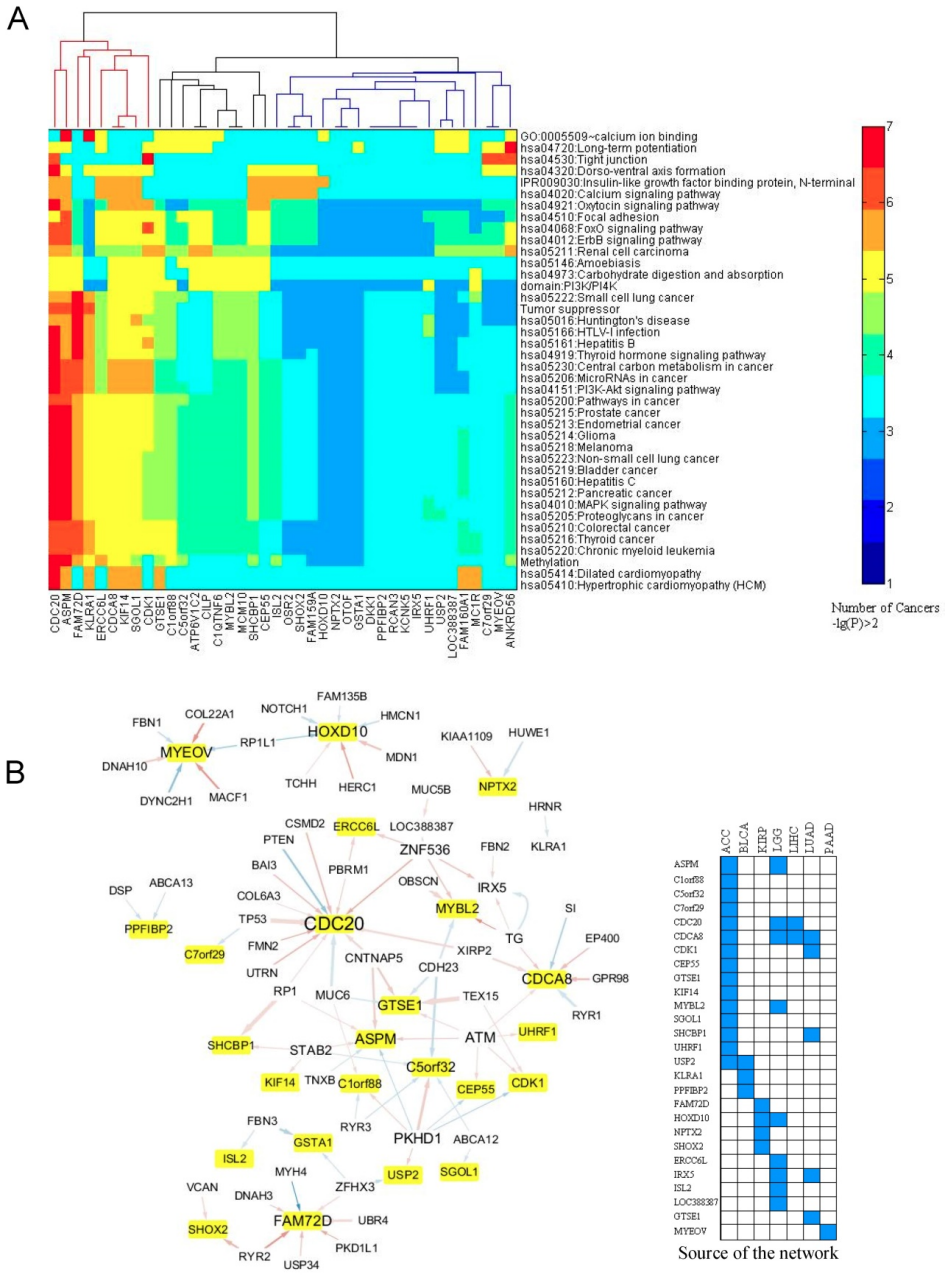
Association between the expression of the prognostic genes and the somatic mutations. **A**. Links between the expression of the prognostic genes and the mutation profile of the top 200 frequently mutated genes. For each prognostic gene, a Chi-squared test was employed to test the association between its expression level and the mutation times of the 200 mutated genes in each cancer. The mutated genes with p ≤ 0.001 were included in an enrichment test for GO term, KEGG pathway, InterPro domain, and SMART mode. A cutoff of p ≤ 0.05 was used to find the enriched terms and pathways. If the criteria were satisfied, a link between the prognostic gene and the mutated pathway (term) was counted once. This was done for each cancer type. The heat map here displays the counts for the links within 29 cancer types. **B**. Generalized linear regression of the expression of the 40 prognostic genes (Table S2) with the mutation profile of the top 200 mutated genes. The network displays the regression results after filtering with p < 0.05 in the Chi-squared test. In the network, the yellow-marked genes are the prognostic genes and the other genes are the mutated genes. A line from the mutated gene to the prognostic gene indicates that the mutation relates to the expression of the prognostic gene. Blue and red lines mean negative and positive effects, respectively. The line width is proportional to the significance (p value of Chi-squared test). A manual validation by literature mining is in Table S4.

We sought to determine the potential links between the associated/mutated pathway and the expression of the 40 prognostic genes identified in Table S2. The computational flowchart is in Fig. S6. The results are shown in Fig. 5. The analysis revealed 3 subgroups (Fig. 5A): 1) the gene expression was affected by many mutated pathways in more than five cancer types. These key genes were *CDC20*, *CDCA8*, *ASPM*, *ERCC6L*, *KLRA1*, *KIF14*, *SGOL1*, and *FAM72D,* 2) the genes whose expression was affected by only a few pathways, such “Focal adhesion”, the “FoxO and ErbB signaling pathways” and “Carbohydrate digestion and absorption”. These genes included *GTS1*, *C1orf88*, *C5orf32*, *ATP6V1C2*, *CLIP*, and *C1QTNF6*. 3) gene whose expression was associated with less than three cancer types. Like *MYEOV*, *ANKRD56*, and *C7orf29* are connected to mutations in the “Tight junction” and “Long-term potentiation” pathways. Mutations occurring in the PI3K/PI4K domain, methylation-related and central carbon metabolism-related genes also showed extensive alteration of the expression levels of the prognostic genes (Fig. 5A).

We further tested the relationships between the mutated genes and the prognostic genes (Fig. 5B). The data showed that *CDC20*, *CDCA8*, and *ASPM* were associated with a greater number (frequency) of mutated genes, while mutations in *PKHD1*, *ATM*, and *ZNF536* were associated with a greater number of prognostic genes. Among the frequently mutated genes, including *TP53* and *PTEN*, showed a strong association to *CDC20* expression (Fig. 5B). As shown in Fig. 5B, mutations in *TG* (thyroglobulin), *EP400*, a component of the NuA4 histone acetyltransferase complex, and *SI* (a sucrase-isomaltase enzyme) showed links with *CDCA8*. Mutations in *CNTNAP5*, which encodes a cell adhesion molecule in the nervous system 28, and mutations in *ATM*, whose product belongs to the PI3/PI4-kinase family and functions as a cell cycle checkpoint kinase, exhibited a link to the prognostic gene *ASPM*. *GTSE1* encodes a protein that is involved in TP53-induced cell cycle arrest in G2/M phase by interfering with microtubule rearrangements 29. We found that mutations in cadherin 23 (*CDH23*), which helps cells stick together, and *TEX15*, which is involved in DNA double-stranded break repair, are linked to *GTSE1* expression. The expression of *MYEOV* is mainly affected by mutations in genes associated with intraflagellar transport (*DYNC2H1*), actin-microtubule interactions and cellular junctions (*MACF1*), myotendinous junctions (*COL22A1*), and calcium-binding microfibrils and glucose homeostasis (*FBN1*).

In order to validate the statistical associations identified above, we thoroughly checked the literatures to verify the links between the prognostic genes and the mutated pathways and genes. The results are summarized in Table S4. TP53-, MAPK-, PI3K- and AKT- related pathways show more associations with the prognostic genes, partly due to that the pathways were extensively studied in the past years (Table S4). On statistical point of view, minor association appeared in the literature, while the biological associations showed a complex scenario. For example, for prognostic gene *CDC20* (Table S4), it was suggested that TP53 or DNA damage induced endogenous TP53 can downregulate *CDC20* transcriptionally. Binding of TP53 at *CDC20* promoter can bring about chromatin remodeling thereby repressing *CDC20* 30, 31. In case of *UHRF1*, its overexpression can affect DNMT1 hence lowers the DNA methylation (Table S4), hence can result into genome instability 32. Also, this overexpression frequently associates to the TP53 mutation 32. In case of *MYEOV* and cell tight junction process (Fig. 5A), although no direct evidence was available, however, an independent experiment suggests that Ras/MEK/ERK pathway controls the junction formation and the inhibition of MEK will decrease the MYEOV expression 33 (Table S4). Overall, the literature mining supports our study as a proof of principle and reliability of our analysis.

To our knowledge, this is the first study which has exclusively elaborated the impact of mutational landscape on the gene expression of prognostic factors.

## Discussion

Genetic and epigenetic features of cancer cells define its phenotypic capacity for the metastasis, however, it is the gene expression signatures which provide better insights about the happening inside the cancer cell. Decoding these signature profiles can not only help in prognostic but also staging the clinical assessments. In this study, we performed comprehensive analysis to identify prognostic genes for overall survival using 29 cancers dataset.

First, we could show that the prognostic genes vary greatly among cancer types. Moreover, cancer with multiple subtypes harbors very fewer prognostic genes. For instance, six subgroups (IDH, K27, G34, RTK I and II, and MES) in GBM 34, five molecular subtypes (Luminal A, Luminal B, Her2 overexpressing, basal, and normal-like) in breast cancers 35 and four subtypes in ESCA 36 can be noticed. These three cancers represent very few prognostic genes (Fig. 1A-B). It is well established that intra-tumor genetic heterogeneity is associated with poorer survival across cancers 37. In this regard, we found a significant association between the inter-tumor expression heterogeneity and the overall survival (Fig. 1C).

Second, considering both survival difference and expression change, we identified 236 prognostic genes. Earlier, it was shown that the cancer-normal expression differentiation is irrelevant to genes survival correlation in multiple cancers and is not helpful in identifying prognostic genes 38.

Herein, we also demonstrate that to identify prognostic genes by comparing gene expression between cancer and control samples is not reliable approach (*DKK1*, Fig. S3). In our analysis, we identified 22 genes concerning prognosis and diagnosis and among them highly significant ones (*CDC20*, *ASPM*, *CDCA8*, *SGOL1*, and *ERCC6L)* were shown to play roles in G2/M processes, such as the spindle assembly checkpoint 21, 22, 39, 40. Therefore, we proposed that the regulation of anaphase of the cell cycle is intimately associated with the patient survival.

Third, we took advantage of mutational data and aim to find their association with relevant prognostic genes. The analysis clearly showed that the mutations in PI3K-AKT, ErbB, and FoxO signaling pathways, in addition to their associated biological processes, can ubiquitously alter the expression of the prognostic genes. In addition, the close association between the prognostic genes that function in anaphase of the cell cycle (*CDC20*, *CDCA8*, *ASPM and GTSE1)* and the mutated genes (*TP53*, *PTEN*, *ATM*, *EP400* and *BAI3)* can be noticed (Fig. 5B). This also indicates about considerable overlapping between gene expression variation and the mutational pathways altered across different tumor types.

Herein, we considered 3 points which are missing in the previous studies. First, both the survival difference P[SV] and the expression level difference (fold change (High group/Low group) (FC(H/L)) ) were considered in identifying the prognostic genes in cancer samples. Second, we also assessed the diagnostic ability of the prognostic genes, which makes it possible to convert the prognostic genes into a clinical test. Third, we statistically linked the prognostic genes to the frequent mutated pathways and the genes, which provides the possible molecular mechanisms for the prognostic genes. In addition, we manually collected some evidences as a proof of in principle supporting our results. Certainly, the translation of our analysis in the cancer genome requires functional elucidations. Importantly, we can hypothesize that these cancer related (mutated) genes are tightly connected and are capable to reshape the genome in one or even multiple cancer types. Taken together, our analysis provided a comprehensive list of genes relevant to prognostic and diagnostic in cancers. Moreover, we revealed the statistically significant link connecting the prognostic cancer genes and the respective cancer related mutations.

## Conclusions

Common prognostic genes with diagnostic capacity for multiple cancers have potential application in planning clinical treatment and in studying cancer mechanism. In this paper, we found 22 genes that have both diagnostic and prognostic capacity and relate to cell cycle and cell division. The universal prognostic genes (*CDC20*, *CDCA8*, *ASPM*, *ERCC6L*, and *GTSE1*), mainly function in anaphase of the cell cycle, especially in the spindle assembly checkpoint. Moreover, we statistically linked the expression of the prognostic genes with the pathways that harbored mutated genes. Our results suggested that the mutations in TP53, PI3K-AKT, ErbB, and FoxO signaling pathways, in addition to their associated biological processes, can ubiquitously alter the expression of the prognostic genes. Briefly, we systematically identified the prognostic genes for overall survival, assessed their diagnostic potential, and linked the genes with mutations.

## Supplementary Material

Supplementary figures and tables.

## References

[1] Uhlen M, Zhang C, Lee S, Sjostedt E, Fagerberg L, Bidkhori G (2017). A pathology atlas of the human cancer transcriptome. Science.

[2] Cardoso F, van't Veer LJ, Bogaerts J, Slaets L, Viale G, Delaloge S (2016). 70-Gene Signature as an Aid to Treatment Decisions in Early-Stage Breast Cancer. N Engl J Med.

[3] Birnbaum DJ, Finetti P, Lopresti A, Gilabert M, Poizat F, Raoul JL (2017). A 25-gene classifier predicts overall survival in resectable pancreatic cancer. BMC Med.

[4] Barrier A, Lemoine A, Boelle PY, Tse C, Brault D, Chiappini F (2005). Colon cancer prognosis prediction by gene expression profiling. Oncogene.

[5] Nault JC, De Reynies A, Villanueva A, Calderaro J, Rebouissou S, Couchy G (2013). A hepatocellular carcinoma 5-gene score associated with survival of patients after liver resection. Gastroenterology.

[6] Lu Y, Lemon W, Liu PY, Yi Y, Morrison C, Yang P (2006). A gene expression signature predicts survival of patients with stage I non-small cell lung cancer. PLoS Med.

[7] Wilson CS, Davidson GS, Martin SB, Andries E, Potter J, Harvey R (2006). Gene expression profiling of adult acute myeloid leukemia identifies novel biologic clusters for risk classification and outcome prediction. Blood.

[8] Navab R, Strumpf D, Bandarchi B, Zhu CQ, Pintilie M, Ramnarine VR (2011). Prognostic gene-expression signature of carcinoma-associated fibroblasts in non-small cell lung cancer. Proc Natl Acad Sci U S A.

[9] Bailey MH, Tokheim C, Porta-Pardo E, Sengupta S, Bertrand D, Weerasinghe A (2018). Comprehensive Characterization of Cancer Driver Genes and Mutations. Cell.

[10] Samur MK (2014). RTCGAToolbox: a new tool for exporting TCGA Firehose data. PLoS One.

[11] Stratford JK, Bentrem DJ, Anderson JM, Fan C, Volmar KA, Marron JS (2010). A six-gene signature predicts survival of patients with localized pancreatic ductal adenocarcinoma. PLoS Med.

[12] Leighton JC Jr, Goldstein LJ (1995). P-glycoprotein in adult solid tumors. Expression and prognostic significance. Hematol Oncol Clin North Am.

[13] Wang Y, Goodison S, Li X, Hu H (2017). Prognostic cancer gene signatures share common regulatory motifs. Sci Rep.

[14] Beyenbach KW, Wieczorek H (2006). The V-type H+ ATPase: molecular structure and function, physiological roles and regulation. J Exp Biol.

[15] Zhang X, Lv QL, Huang YT, Zhang LH, Zhou HH (2017). Akt/FoxM1 signaling pathway-mediated upregulation of MYBL2 promotes progression of human glioma. J Exp Clin Cancer Res.

[16] Guan Z, Cheng W, Huang D, Wei A (2018). High MYBL2 expression and transcription regulatory activity is associated with poor overall survival in patients with hepatocellular carcinoma. Curr Res Transl Med.

[17] Fan X, Wang Y, Jiang T, Cai W, Jin Y, Niu Y (2018). B-Myb Mediates Proliferation and Migration of Non-Small-Cell Lung Cancer via Suppressing IGFBP3. Int J Mol Sci.

[18] Hong SA, Yoo SH, Lee HH, Sun S, Won HS, Kim O (2018). Prognostic value of Dickkopf-1 and ss-catenin expression in advanced gastric cancer. BMC Cancer.

[19] Skiriute D, Vaitkiene P, Asmoniene V, Steponaitis G, Deltuva VP, Tamasauskas A (2013). Promoter methylation of AREG, HOXA11, hMLH1, NDRG2, NPTX2 and Tes genes in glioblastoma. J Neurooncol.

[20] Carlson MR, Pope WB, Horvath S, Braunstein JG, Nghiemphu P, Tso CL (2007). Relationship between survival and edema in malignant gliomas: role of vascular endothelial growth factor and neuronal pentraxin 2. Clin Cancer Res.

[21] Izawa D, Pines J (2011). How APC/C-Cdc20 changes its substrate specificity in mitosis. Nat Cell Biol.

[22] Gassmann R, Carvalho A, Henzing AJ, Ruchaud S, Hudson DF, Honda R (2004). Borealin: a novel chromosomal passenger required for stability of the bipolar mitotic spindle. J Cell Biol.

[23] Musa J, Aynaud MM, Mirabeau O, Delattre O, Grunewald TG (2017). MYBL2 (B-Myb): a central regulator of cell proliferation, cell survival and differentiation involved in tumorigenesis. Cell Death Dis.

[24] Diril MK, Ratnacaram CK, Padmakumar VC, Du T, Wasser M, Coppola V (2012). Cyclin-dependent kinase 1 (Cdk1) is essential for cell division and suppression of DNA re-replication but not for liver regeneration. Proc Natl Acad Sci U S A.

[25] Zhu C, Zhao J, Bibikova M, Leverson JD, Bossy-Wetzel E, Fan JB (2005). Functional analysis of human microtubule-based motor proteins, the kinesins and dyneins, in mitosis/cytokinesis using RNA interference. Mol Biol Cell.

[26] Corson TW, Zhu CQ, Lau SK, Shepherd FA, Tsao MS, Gallie BL (2007). KIF14 messenger RNA expression is independently prognostic for outcome in lung cancer. Clin Cancer Res.

[27] Stanietsky N, Simic H, Arapovic J, Toporik A, Levy O, Novik A (2009). The interaction of TIGIT with PVR and PVRL2 inhibits human NK cell cytotoxicity. Proc Natl Acad Sci U S A.

[28] Spiegel I, Salomon D, Erne B, Schaeren-Wiemers N, Peles E (2002). Caspr3 and caspr4, two novel members of the caspr family are expressed in the nervous system and interact with PDZ domains. Mol Cell Neurosci.

[29] Xu T, Ma M, Chi Z, Si L, Sheng X, Cui C (2018). High G2 and S-phase expressed 1 expression promotes acral melanoma progression and correlates with poor clinical prognosis. Cancer Sci.

[30] Banerjee T, Nath S, Roychoudhury S (2009). DNA damage induced p53 downregulates Cdc20 by direct binding to its promoter causing chromatin remodeling. Nucleic Acids Research.

[31] Kidokoro T, Tanikawa C, Furukawa Y, Katagiri T, Nakamura Y, Matsuda K (2008). CDC20, a potential cancer therapeutic target, is negatively regulated by p53. Oncogene.

[32] Mudbhary R, Hoshida Y, Chernyavskaya Y, Jacob V, Villanueva A, Fie MI (2014). UHRF1 Overexpression Drives DNA Hypomethylation and Hepatocellular Carcinoma. Cancer Cell.

[33] Joanne Durgan1 GT, Matthew S Walters, Oliver Florey, Anja Schmidt, Vanessa Arbelaez, Neal Rosen, Ronald G Crystal, Alan Hall1 (2015). SOS1 and Ras regulate epithelial tight junction formation in the human airway through EMP1. EMBO reports.

[34] Sturm D, Witt H, Hovestadt V, Khuong-Quang DA, Jones DT, Konermann C (2012). Hotspot mutations in H3F3A and IDH1 define distinct epigenetic and biological subgroups of glioblastoma. Cancer Cell.

[35] Dai X, Li T, Bai Z, Yang Y, Liu X, Zhan J (2015). Breast cancer intrinsic subtype classification, clinical use and future trends. Am J Cancer Res.

[36] Cancer Genome Atlas Research N, Analysis Working Group (2017). Asan U, Agency BCC, Brigham, Women's H, Broad I, et al. Integrated genomic characterization of oesophageal carcinoma. Nature.

[37] Morris LG, Riaz N, Desrichard A, Senbabaoglu Y, Hakimi AA, Makarov V (2016). Pan-cancer analysis of intratumor heterogeneity as a prognostic determinant of survival. Oncotarget.

[38] An N, Yu Z, Yang X (2018). Expression Differentiation Is Not Helpful in Identifying Prognostic Genes Based on TCGA Datasets. Mol Ther Nucleic Acids.

[39] Jiang K, Rezabkova L, Hua S, Liu Q, Capitani G, Altelaar AFM (2017). Microtubule minus-end regulation at spindle poles by an ASPM-katanin complex. Nat Cell Biol.

[40] Baumann C, Korner R, Hofmann K, Nigg EA (2007). PICH, a centromere-associated SNF2 family ATPase, is regulated by Plk1 and required for the spindle checkpoint. Cell.

