# Supplementary material for: Prognostic gene expression signature revealed the involvement of mutational pathways in cancer genome
Source: J Cancer. 2020 May 18;11(15):4510–20. doi: 10.7150/jca.40237 (PMC7255374; doi:10.7150/jca.40237)
Supplement: Supplementary file 1 — Supplementary figures and tables. [file jcav11p4510s1.pdf]

Supplementary Information

Prognostic gene expression signature revealed the involvement of mutational pathways in cancer genome

Hongde Liu[1*†], Huamei Li[1†], Kun Luo[2], Amit Sharma[3], Xiao Sun[1]

1State Key Laboratory of Bioelectronics, School of Biological Science & Medical Engineering, Southeast University, Nanjing 210096, China;
2Department of Neurosurgery, Xinjiang Evidence-Based Medicine Research Institute, First Affiliated Hospital of Xinjiang Medical University, Urumqi 830054, China;
3Department of Ophthalmology, University Hospital Bonn, Germany.

E-mails:
Hongde Liu: liuhongde@seu.edu.cn (HDL)
Huamei Li: li_hua_mei@163.com (HML)
Kun Luo: luokun_2822@sohu.com (KL)
Amit Sharma: Amit.Sharma@ukbonn.de (AS)
Xiao Sun: xsun@seu.edu.cn (XS)

*correspondence:
Hongde Liu
liuhongde@seu.edu.cn

†Contributed equally to this article

Materials and methods

Datasets

Gene expression data, survival data, and mutation data used in this study were retrieved from TCGA project from the initial release of Genomic Data Commons (GDC) in October 2016 (https://portal.gdc.cancer.gov/) using RTCGAToolbox[1]. A total of 9523 samples across 29 tumor types were downloaded, including 8811 tumor tissues and 712 non-tumor tissues. Microarray-based gene expression data (gene expression omnibus ID: GSE21501 for pancreatic cancer [2]) were retrieved for validating the expression markers for survival.

Identification of the prognostic genes

Genes whose expression is associated with a differential overall survival were identified with a log-rank test in a Kaplan–Meier survival model. In each cancer type, for each gene, patients were classified into two groups using the expression median of the gene as a cutoff. The two groups were named the high-expression group (H) and the low-expression group (L), depending on whether the expression level was higher or lower than the median, respectively. The survival difference was tested in the two groups. In identifying, we considered both survival difference and the expression change between the two groups. First, the criteria of a survival difference $P[SV]\leq10^{-3}$ and a fold change of expression $(FC(H/L))\geq2$ were applied to 20,531 genes for 29 cancer types, resulting in a list of 236 genes by choosing the top ten genes. Then, stricter criteria, $P[SV]\leq10^{-6}$ and $FC[H/L]>4$ were applied to the 236 genes, resulting in a list of 40 genes. Finally, we were interested in the possibility of the prognostic genes acting as markers to diagnose cancer and normal tissues. Thus, the expression difference between cancer (C) and control (N) tissues was tested. The area under the curve (AUC) of a receiver operating characteristic (ROC) curve and the expression fold change (FC(C/N)) between the cancer and normal tissues were employed to indicate the difference, namely $AUC\geq0.8$ and $|\log_2[FC(C/N)]|\geq2$, resulting in a list of 22 genes.

We also identified prognostic genes another way, by examining the Pearson correlation coefficient between gene expression and survival time among the population. Candidate genes were those with a large positive or negative correlation.

Analysis of association between immunoregulation-related gene expression and survival time

Fifteen immunoregulation-related genes were considered, including *PDL-1*, *PDL-2*, and *PVR*. PDL-1 and PDL-2 are ligands of programmed death-1 (PD-1), which is involved in the inhibition of T cells [3], and PVR is a ligand of TIGIT. The binding of the two inhibits NK

cell cytotoxicity [4].

Analysis of the mutations in cancers

For each gene, mutations were counted in all cancers. A mutation rate (frequency) of the gene is the ratio of the total mutations in the gene to the number of all patients in all cancers. The top 200 frequently mutated genes were used in a regression model for the expression of the prognostic genes to examine what kind of mutations contributed to the expression levels of the prognostic genes (see below). Given that the mutation count is related to both gene length and gene expression [5, 6], the mutation rate was normalized by dividing the rate by gene length [Kbp].

We investigated the relationship between the mutation counts and the survival time. The survival time was truncated at a survival probability of 60%. We also tested the relationship between the normalized mutation rate and the expression level.

Regression for the expression of the prognostic genes with the mutation counts

The 40 prognostic genes, which were identified with $P[SV]\leq10^{-6}$ and $FC[H/L]\geq4$, and the top 200 frequently mutated genes, were included in this section of analysis (Fig. S6). Firstly, the prognostic genes were aligned to the pathways enriched for the mutated genes and GO terms. For each prognostic gene, the dependence between its expression and the mutation counts of the 200 mutated genes were tested with a chi-squared (χ^2) test for each cancer. The mutated genes with $p \leq 0.001$ (χ^2 test) were included in an enrichment analysis on GO terms, KEGG pathways, InterPro domains, and SMART modes, based on a hypergeometric distribution or Fisher’s exact test. A cutoff of $p \leq 0.05$ was used to find the enriched terms and pathways. Upon satisfaction of those criteria, a link between the prognostic gene and the mutated pathway (terms) was counted. This was done for all cancers to see how many cancer types shared the link.

Secondly, we carried out a generalized linear regression of the expression of the prognostic gene with the mutation counts of the top 200 frequently mutated genes for each cancer type. The regression generated a set of parameters indicating the contribution of the mutation in explaining the expression level of the prognostic gene. Only mutated genes with a significant parameter were used to construct a network.

Enrichment analysis

The enrichment analysis for the 236 prognostic genes and the top 200 frequently mutated genes was carried out with DAVID [7].

Other analysis in the work

The activation status of pathways was assessed with SPIA [8]. Survival analysis and the survival comparison were carried out by an inhouse Matlab program.

Abbreviation for cancer types

LAML: Acute Myeloid Leukemia;

ACC: Adrenocortical carcinoma;

BLCA: Bladder Urothelial Carcinoma;

LGG: Brain Lower Grade Glioma;

BRCA: Breast invasive carcinoma;

CESC: Cervical squamous cell carcinoma and endocervical adenocarcinoma;

CHOL: Cholangiocarcinoma;

LCML: Chronic Myelogenous Leukemia;

COAD: Colon adenocarcinoma;

ESCA: Esophageal carcinoma;

GBM: Glioblastoma multiforme;

HNSC: Head and Neck squamous cell carcinoma;

KICH: Kidney Chromophobe;

KIRC: Kidney renal clear cell carcinoma;

KIRP: Kidney renal papillary cell carcinoma;

LIHC: Liver hepatocellular carcinoma;

LUAD: Lung adenocarcinoma;

LUSC: Lung squamous cell carcinoma;

DLBC: Lymphoid Neoplasm Diffuse Large B-cell Lymphoma;

MESO: Mesothelioma;

OV: Ovarian serous cystadenocarcinoma;

PAAD: Pancreatic adenocarcinoma;

PCPG: Pheochromocytoma and Paraganglioma;

PRAD: Prostate adenocarcinoma;

STAD: Stomach adenocarcinoma;

TGCT: Testicular Germ Cell Tumors;

THYM: Thymoma;

THCA: Thyroid carcinoma;

UCS: Uterine Carcinosarcoma;

UCEC: Uterine Corpus Endometrial Carcinoma.

References

1. Samur MK. RTCGAToolbox: a new tool for exporting TCGA Firehose data. PLoS One. 2014; 9: e106397.
2. Stratford JK, Bentrem DJ, Anderson JM, Fan C, Volmar KA, Marron JS, et al. A six-gene signature predicts survival of patients with localized pancreatic ductal adenocarcinoma. PLoS Med. 2010; 7: e1000307.
3. Xu-Monette ZY, Zhou J, Young KH. PD-1 expression and clinical PD-1 blockade in B-cell lymphomas. Blood. 2018; 131: 68-83.
4. Stanietsky N, Simic H, Arapovic J, Toporik A, Levy O, Novik A, et al. The interaction of TIGIT with PVR and PVRL2 inhibits human NK cell cytotoxicity. Proc Natl Acad Sci U S A. 2009; 106: 17858-63.
5. Bailey MH, Tokheim C, Porta-Pardo E, Sengupta S, Bertrand D, Weerasinghe A, et al. Comprehensive Characterization of Cancer Driver Genes and Mutations. Cell. 2018; 174: 1034-5.
6. Lawrence MS, Stojanov P, Polak P, Kryukov GV, Cibulskis K, Sivachenko A, et al. Mutational heterogeneity in cancer and the search for new cancer-associated genes. Nature. 2013; 499: 214-8.
7. Huang da W, Sherman BT, Lempicki RA. Systematic and integrative analysis of large gene lists using DAVID bioinformatics resources. Nat Protoc. 2009; 4: 44-57.
8. Tarca AL, Draghici S, Khatri P, Hassan SS, Mittal P, Kim JS, et al. A novel signaling pathway impact analysis. Bioinformatics. 2009; 25: 75-82.

Supplementary Tables

Table S1 Two hundred and thirty six genes with prognostic capacity.

The prognostic genes identified at criteria of P[SV]≤10^{-3} and FC(H/L)≥2, selecting the top ten genes for each cancer type. There were a total of 236 genes in 29 cancer types. Here, in each cancer type, for each gene, patients are classified into high (H) and low (L) expression groups using the expression median of the gene as a cutoff. The survival difference (P[SV]) was tested between the two groups with a log-rank test. FC(H/L) means the fold change of average expression between the two groups. Chi-square is the value of χ^2 in the log-rank test, T-middle-H and T-middle-L are the time at 50% survival probability for the high- and low-expression groups, respectively, and mean-H and mean-L are the mean expression in the high- and low-expression groups, respectively. The gene symbol and cell type are the official symbol of the prognostic gene and the corresponding cancer type, respectively. "NaN" means within the observed time, more than 50% of the patients survived, thus there is no data for "T-middle-H" and "T-middle-L".

Gene Symbol | Entrez gene ID	Chi-Square	−lg(P[SV])	T-middle-H (days)	T-middle-L (days)	Mean-H (FPKM)	mean-L (FPKM)	log2(FC(H/L)	Cancer type
C1orf88|128344	34.03	8.27	NaN	1194.50	298.17	13.72	4.44	ACC
FAM72D|728833	30.67	7.52	1105.08	NaN	94.07	22.45	2.07	ACC
ASPM|259266	30.54	7.48	1096.17	NaN	469.85	56.66	3.05	ACC
BDH2|56898	28.81	7.10	NaN	1029.01	3534.80	1020.79	1.79	ACC
GSTA1|2938	28.66	7.06	NaN	1197.21	52294.74	1292.30	5.34	ACC
PPFIBP2|8495	28.21	6.96	NaN	1171.11	394.52	85.86	2.20	ACC
USP2|9099	28.10	6.94	NaN	1171.07	988.80	120.45	3.04	ACC
C5orf32|84418	27.60	6.83	NaN	1194.73	8730.61	2008.55	2.12	ACC
PPRC1|23082	27.51	6.81	1171.04	NaN	1316.74	656.32	1.00	ACC

CDK1|983	27.28	6.75	1096.16	NaN	1269.00	188.31	2.75	ACC
BCL2L14|79370	26.50	6.58	2790.04	665.08	137.79	15.88	3.12	BLCA
ANXA1|301	21.13	5.37	680.34	2009.13	15764.75	2200.09	2.84	BLCA
HTRA1|5654	20.29	5.18	690.30	2964.01	4339.03	816.00	2.41	BLCA
AOC2|314	20.14	5.14	2886.02	685.07	165.17	17.54	3.24	BLCA
APOL2|23780	19.95	5.10	2964.01	674.11	3293.06	974.26	1.76	BLCA
AGER|177	19.64	5.03	2703.04	700.53	177.04	48.41	1.87	BLCA
CSAD|51380	19.64	5.03	3011.01	706.62	609.47	175.53	1.80	BLCA
EMP1|2012	19.33	4.96	719.14	NaN	5034.80	927.33	2.44	BLCA
PPFIBP2|8495	19.29	4.95	2330.01	700.25	2795.15	449.13	2.64	BLCA
KLRA1|10748	19.25	4.94	2423.02	700.10	57.46	12.68	2.18	BLCA
FAM159A|348378	15.67	4.12	NaN	4361.05	19.29	3.95	2.29	BRCA
TRAT1|50852	15.60	4.11	NaN	4233.08	61.66	4.96	3.64	BRCA
TXNDC6|347736	15.55	4.10	NaN	4285.01	7.91	1.91	2.05	BRCA
TMEM65|157378	14.79	3.92	5156.01	NaN	907.39	323.43	1.49	BRCA
PGK1|5230	13.54	3.63	7126.00	NaN	12785.40	5836.47	1.13	BRCA
TP53AIP1|63970	13.49	3.62	NaN	4361.02	23.58	2.49	3.24	BRCA
CYP24A1|1591	13.25	3.57	NaN	NaN	143.64	0.76	7.56	BRCA
PCSK6|5046	13.18	3.55	NaN	4361.05	2285.58	381.81	2.58	BRCA

FAM166B|730112	13.06	3.52	NaN	NaN	30.44	2.25	3.76	BRCA
RAC2|5880	12.94	3.49	NaN	4354.03	1293.97	370.63	1.80	BRCA
PSMA8|143471	21.42	5.43	NaN	2052.04	0.97	0.00	7.82	CESC
RBM38|55544	19.82	5.07	NaN	2234.03	1924.46	762.89	1.33	CESC
TREX1|11277	19.12	4.91	NaN	2669.01	1004.79	412.51	1.28	CESC
TAF1A|9015	17.50	4.54	3039.33	NaN	175.30	84.17	1.06	CESC
BTNL8|79908	17.39	4.52	NaN	2520.01	36.16	0.28	7.00	CESC
MLLT4|4301	17.06	4.44	2114.01	NaN	2592.57	1206.00	1.10	CESC
ZNF827|152485	16.68	4.35	2520.01	NaN	162.92	38.71	2.07	CESC
RCAN3|11123	16.40	4.29	3039.05	NaN	193.80	84.21	1.20	CESC
PRSS36|146547	16.05	4.21	NaN	3043.49	222.21	44.61	2.32	CESC
SNHG9|735301	16.01	4.20	NaN	2394.01	98.74	26.20	1.91	CESC
ATP13A3|79572	11.09	3.06	NaN	602.07	5094.96	2411.55	1.08	CHOL
SCN9A|6335	10.94	3.03	627.71	1542.01	465.20	22.76	4.35	CHOL
TNNT2|7139	19.79	5.06	1856.08	NaN	18.25	1.16	3.98	COAD
ENO3|2027	17.29	4.49	1915.02	NaN	66.01	22.21	1.57	COAD
GSR|2936	16.77	4.37	NaN	1849.04	2129.33	887.82	1.26	COAD
DFNB59|494513	16.45	4.30	1856.16	NaN	16.10	5.41	1.57	COAD
ATOH1|474	16.04	4.21	NaN	1881.05	332.04	17.65	4.23	COAD

FLJ33360|401172	15.76	4.14	1674.03	NaN	2.37	0.19	3.65	COAD
CAPRIN2|65981	15.62	4.11	2131.41	NaN	339.94	142.98	1.25	COAD
EGFL8|80864	15.42	4.07	1849.08	NaN	120.34	46.17	1.38	COAD
FAM160A1|729830	15.30	4.04	NaN	1977.08	152.14	69.48	1.13	COAD
ATP8B1|5205	14.83	3.93	NaN	1829.12	5249.45	2439.42	1.11	COAD
C11orf74|119710	13.34	3.59	5980.00	1739.00	61.89	23.51	1.40	DLBC
VLDLR|7436	11.90	3.25	5980.00	3333.01	27.87	7.94	1.81	DLBC
ENO3|2027	11.48	3.15	2131.02	5980.00	262.13	74.63	1.81	DLBC
ATP1A3|478	11.25	3.10	NaN	1739.00	303.57	83.85	1.86	DLBC
TRAIP|10293	11.20	3.09	5980.00	3394.02	567.04	224.41	1.34	DLBC
DMRTA2|63950	11.04	3.05	3394.02	5980.00	5.31	0.03	7.44	DLBC
CCDC13|152206	10.97	3.03	5980.01	NaN	30.32	8.75	1.79	DLBC
TMC3|342125	10.84	3.00	5980.00	3394.03	3.68	0.86	2.10	DLBC
GRPEL2|134266	12.49	3.39	558.07	1168.01	1009.92	429.96	1.23	ESCA
GXYLT1|283464	12.13	3.30	559.48	1263.01	646.88	290.49	1.16	ESCA
ANGPT2|285	11.96	3.27	563.12	1361.05	638.42	196.36	1.70	ESCA
GPER|2852	11.21	3.09	961.03	553.58	140.86	25.09	2.49	ESCA
TRIM47|91107	11.10	3.06	882.05	712.18	2005.45	741.15	1.44	ESCA
NBL1|4681	10.99	3.04	1441.18	558.12	3607.73	1622.67	1.15	ESCA

KIAA1430|57587	10.83	3.00	1361.18	610.67	1496.69	733.86	1.03	ESCA
OPN3|23596	16.37	4.28	343.08	480.24	409.81	192.69	1.09	GBM
FOXP3|50943	16.15	4.23	348.58	480.29	18.45	4.78	1.95	GBM
MICALL2|79778	13.84	3.70	342.25	470.15	1140.54	412.22	1.47	GBM
LOC100132111|100132111	13.23	3.56	335.16	470.46	8.64	0.90	3.26	GBM
TSHZ2|128553	12.56	3.41	323.67	485.38	107.02	19.89	2.43	GBM
MLPH|79083	12.55	3.40	331.41	468.08	81.14	2.84	4.84	GBM
HIST3H2A|92815	12.12	3.30	470.14	343.09	170.55	27.00	2.66	GBM
LRRC61|65999	11.97	3.27	335.14	489.11	337.54	41.68	3.02	GBM
C7orf29|113763	11.82	3.23	357.59	470.07	132.49	17.10	2.95	GBM
RARRES2|5919	11.74	3.21	381.63	443.63	2118.73	310.42	2.77	GBM
DKK1|22943	27.75	6.86	985.60	2083.03	777.62	60.78	3.68	HNSC
EVPLL|645027	23.71	5.95	2727.08	1075.46	125.63	11.43	3.46	HNSC
FGD3|89846	21.15	5.37	2135.55	954.04	420.83	139.55	1.59	HNSC
PITPNM3|83394	20.15	5.14	2562.01	1134.53	998.09	354.49	1.49	HNSC
TMSB4Y|9087	19.92	5.09	2703.04	1037.68	34.51	0.82	5.40	HNSC
STC2|8614	19.73	5.05	1082.21	2886.46	1148.35	227.33	2.34	HNSC
GNA12|2768	18.04	4.67	1007.17	2064.09	2969.39	1417.35	1.07	HNSC
CHGB|1114	17.94	4.64	1079.11	2641.13	1900.89	6.00	8.31	HNSC

FGFR2|2263	17.74	4.60	2083.17	1183.26	1744.29	456.12	1.94	HNSC
ZNF266|10781	17.56	4.56	2641.02	1131.73	707.57	335.23	1.08	HNSC
MYH14|79784	13.59	3.64	NaN	NaN	5989.56	2920.56	1.04	KICH
DBNDD1|79007	13.54	3.63	NaN	NaN	1003.46	302.97	1.73	KICH
FAM71E1|112703	12.72	3.44	NaN	NaN	352.79	127.90	1.46	KICH
BAHCC1|57597	12.53	3.40	NaN	NaN	763.14	362.99	1.07	KICH
BOK|666	12.29	3.34	NaN	NaN	3001.33	809.79	1.89	KICH
CA11|770	12.21	3.32	NaN	NaN	501.18	180.97	1.47	KICH
CLDN3|1365	12.21	3.32	NaN	NaN	403.97	49.27	3.04	KICH
C15orf56|644809	12.15	3.31	NaN	NaN	139.20	43.66	1.67	KICH
NAPRT1|93100	11.97	3.27	NaN	NaN	1241.51	481.97	1.37	KICH
KCNK5|8645	11.95	3.26	NaN	NaN	183.04	20.11	3.19	KICH
DONSON|29980	57.33	13.43	1658.90	NaN	233.83	103.36	1.18	KIRC
ANKRD56|345079	51.42	12.13	NaN	1714.10	267.58	73.83	1.86	KIRC
MC1R|4157	51.23	12.09	1906.27	NaN	188.18	49.91	1.91	KIRC
PRSS53|339105	49.90	11.79	1889.35	NaN	120.51	28.78	2.07	KIRC
SLC4A5|57835	49.82	11.77	1955.21	NaN	214.98	56.25	1.93	KIRC
FKBP11|51303	49.62	11.73	1714.12	NaN	862.36	246.74	1.81	KIRC
STAT2|6773	49.57	11.72	1714.12	NaN	3077.99	1466.47	1.07	KIRC

TMEM44|93109	48.28	11.43	1906.22	NaN	696.40	233.12	1.58	KIRC
ATP6V1C2|245973	48.21	11.42	1955.38	NaN	307.73	39.59	2.96	KIRC
OTOF|9381	47.78	11.32	1641.94	NaN	9.07	1.42	2.68	KIRC
NPTX2|4885	26.43	6.56	2649.03	NaN	145.50	4.37	5.06	KIRP
DLEU2|8847	22.07	5.58	2839.04	NaN	28.36	7.76	1.87	KIRP
SPAG5|10615	21.57	5.47	NaN	NaN	433.99	96.26	2.17	KIRP
SLC4A5|57835	21.16	5.37	NaN	NaN	137.59	42.54	1.69	KIRP
FAM72D|728833	20.67	5.26	NaN	NaN	22.66	4.49	2.33	KIRP
SHOX2|6474	20.54	5.23	2839.26	NaN	15.31	0.20	6.24	KIRP
CKS1B|1163	19.15	4.92	NaN	NaN	369.23	182.09	1.02	KIRP
MTHFD2|10797	18.39	4.74	2839.06	NaN	529.88	115.37	2.20	KIRP
HOXD10|3236	17.90	4.63	2839.05	NaN	145.51	4.92	4.89	KIRP
IDO1|3620	17.72	4.59	NaN	NaN	142.26	17.91	2.99	KIRP
TREML2|79865	17.86	4.62	306.16	945.06	474.77	132.91	1.84	LAML
SYCE1|93426	17.07	4.44	1277.04	306.05	24.30	0.17	7.17	LAML
NUP210|23225	16.63	4.34	275.04	945.04	13459.91	6378.93	1.08	LAML
CCND3|896	15.75	4.14	304.46	945.08	4427.51	1999.36	1.15	LAML
MYH15|22989	14.83	3.93	306.10	915.04	12.09	1.81	2.74	LAML
TBX1|6899	13.01	3.51	1277.02	335.05	66.02	6.11	3.43	LAML

PDE3B|5140	12.94	3.49	882.03	339.63	3180.71	1109.56	1.52	LAML
LOC646762|646762	12.92	3.49	854.20	363.55	1412.66	637.46	1.15	LAML
LNP1|348801	12.88	3.48	854.03	305.43	44.24	18.60	1.25	LAML
PLA2G4A|5321	12.79	3.46	335.07	1404.84	636.15	202.96	1.65	LAML
ISL2|64843	55.23	12.97	1519.24	4113.01	28.54	0.42	6.08	LGG
OSR2|116039	51.52	12.15	1868.15	4445.02	28.25	0.41	6.12	LGG
WEE1|7465	51.47	12.14	1650.11	3733.00	575.56	133.45	2.11	LGG
HOXC4|3221	51.05	12.05	1578.23	4229.01	77.75	6.01	3.69	LGG
IQGAP2|10788	48.89	11.57	1828.02	4113.02	510.37	61.24	3.06	LGG
SYDE1|85360	46.48	11.03	1915.15	4445.01	559.36	225.37	1.31	LGG
GNG12|55970	46.06	10.94	1868.14	4445.07	1830.55	351.47	2.38	LGG
CRY2|1408	45.09	10.73	4412.08	1578.01	2916.12	1372.52	1.09	LGG
LOC388387|388387	43.54	10.38	4229.01	1631.04	8.52	0.78	3.44	LGG
TMEM106C|79022	42.43	10.14	1666.09	3761.03	895.34	388.41	1.20	LGG
CDC20|991	24.23	6.07	1098.04	2513.23	969.63	136.48	2.83	LIHC
SOX11|6664	21.72	5.50	1135.15	2532.17	13.29	0.18	6.19	LIHC
LEPRE1|64175	19.84	5.08	1219.84	2513.49	1577.99	783.70	1.01	LIHC
SLC25A15|10166	18.41	4.75	2513.04	1168.66	5565.80	1158.56	2.26	LIHC
HN1|51155	18.41	4.75	1242.10	2752.01	2124.71	673.25	1.66	LIHC

C22orf9|23313	18.00	4.66	1386.13	2752.02	2862.62	1158.38	1.31	LIHC
SOCS2|8835	17.70	4.59	2513.05	1363.22	793.48	132.21	2.59	LIHC
CDCA8|55143	17.57	4.56	1135.11	2515.20	420.19	88.78	2.24	LIHC
C17orf44|284029	16.92	4.41	NaN	1363.11	131.00	44.33	1.56	LIHC
LOC728989|728989	16.62	4.34	2456.03	1135.55	14.87	1.75	3.09	LIHC
SFTPB|6439	22.03	5.57	2261.09	1246.09	353773.70	32059.70	3.46	LUAD
CYP17A1|1586	21.38	5.42	2973.02	1258.47	74.74	0.49	7.26	LUAD
C1QTNF6|114904	21.29	5.40	1216.12	2616.02	1076.51	309.96	1.80	LUAD
IRX5|10265	20.43	5.21	2368.09	1215.14	606.54	119.10	2.35	LUAD
EIF5AL1|143244	20.34	5.19	1194.05	2618.74	2105.54	1051.87	1.00	LUAD
BEX4|56271	20.14	5.14	2174.08	1194.79	910.25	294.98	1.63	LUAD
ERO1LB|56605	19.90	5.09	2676.25	1130.13	1024.71	182.26	2.49	LUAD
STEAP1|26872	19.74	5.05	1209.10	1778.08	914.29	153.72	2.57	LUAD
MYLIP|29116	19.46	4.99	2974.25	1215.10	1143.04	507.76	1.17	LUAD
GTSE1|51512	19.30	4.95	1209.30	1798.05	362.16	90.87	1.99	LUAD
KIAA0408|9729	13.95	3.73	1154.18	2336.33	38.52	0.39	6.63	LUSC
PARVA|55742	13.87	3.71	1114.18	2336.03	1644.34	786.68	1.06	LUSC
CST3|1471	13.15	3.54	1072.36	2170.01	5312.13	2453.99	1.11	LUSC
CD151|977	12.56	3.41	1107.62	2284.13	6057.86	2563.09	1.24	LUSC

PAPPA|5069	12.45	3.38	1311.05	2378.05	355.22	42.72	3.06	LUSC
EDEM1|9695	12.31	3.35	1163.80	2336.09	2595.57	1277.41	1.02	LUSC
JPH1|56704	12.29	3.34	2284.02	1067.16	575.26	165.88	1.79	LUSC
DHDPSL|112817	12.15	3.31	2271.32	1150.35	77.90	10.75	2.86	LUSC
CHST15|51363	12.13	3.30	1111.03	2271.14	1767.48	714.35	1.31	LUSC
NT5E|4907	12.09	3.30	1115.26	2080.18	1059.04	109.42	3.27	LUSC
SHCBP1|79801	39.04	9.38	333.09	826.16	490.56	161.45	1.60	MESO
ORC6L|23594	37.92	9.13	333.20	823.38	214.56	74.21	1.53	MESO
UHRF1|29128	37.75	9.09	350.48	826.25	533.75	139.68	1.93	MESO
KIF14|9928	37.45	9.03	350.29	823.44	253.86	56.87	2.16	MESO
CEP55|55165	36.61	8.84	359.80	823.38	774.82	197.68	1.97	MESO
ERCC6L|54821	35.49	8.59	333.08	823.43	98.40	27.10	1.86	MESO
MYBL2|4605	35.38	8.57	333.09	823.43	1565.73	247.15	2.66	MESO
GINS1|9837	33.48	8.14	350.17	823.11	370.86	152.64	1.28	MESO
SGOL1|151648	33.18	8.08	361.09	823.75	109.80	31.05	1.82	MESO
MCM10|55388	32.97	8.03	360.25	795.04	177.76	42.20	2.07	MESO
C1orf115|79762	12.48	3.39	1688.63	1082.24	511.00	168.33	1.60	OV
DYDC2|84332	12.26	3.34	1646.02	1105.65	142.99	29.30	2.29	OV
CDK14|5218	12.17	3.31	1155.62	1485.54	883.91	303.47	1.54	OV

EMP1|2012	11.90	3.25	1164.43	1573.35	4265.43	1257.93	1.76	OV
SNORD15B|114599	10.95	3.03	1213.08	1583.02	49.86	1.38	5.17	OV
MYEOV|26579	24.43	6.11	460.38	1323.11	1934.68	312.40	2.63	PAAD
EPS8|2059	21.36	5.42	470.37	1021.03	3150.64	1541.36	1.03	PAAD
PHLDB3|653583	20.24	5.17	2016.23	476.55	359.87	149.31	1.27	PAAD
ZNF596|169270	19.83	5.07	1037.08	470.75	66.40	30.58	1.12	PAAD
SLURP1|57152	19.47	4.99	468.08	1326.44	11.15	0.02	9.04	PAAD
FAM83A|84985	19.44	4.98	470.43	1216.05	1764.47	109.34	4.01	PAAD
ABHD8|79575	18.99	4.88	1037.40	476.22	402.92	196.10	1.04	PAAD
TMEM175|84286	17.84	4.62	998.02	525.40	748.29	321.31	1.22	PAAD
MET|4233	17.32	4.50	485.18	1037.08	5350.07	1722.23	1.64	PAAD
MPZL2|10205	17.01	4.43	502.76	720.09	2932.66	1051.46	1.48	PAAD
DUSP27|92235	10.96	3.03	NaN	3534.08	6.38	1.01	2.66	PCPG
C15orf56|644809	11.16	3.08	NaN	3479.05	34.64	9.46	1.87	PRAD
KIAA0319|9856	10.94	3.03	NaN	NaN	39.13	5.85	2.74	PRAD
NTNG1|22854	18.93	4.87	754.68	NaN	21.12	0.74	4.84	STAD
MFGE8|4240	17.75	4.60	754.13	NaN	3628.64	1327.70	1.45	STAD
SOX18|54345	16.23	4.25	752.15	NaN	852.01	271.31	1.65	STAD
SOX17|64321	16.05	4.21	785.27	NaN	144.84	38.95	1.89	STAD

VASN|114990	16.03	4.21	865.11	NaN	897.42	280.52	1.68	STAD
MLEC|9761	15.98	4.19	NaN	779.56	11499.76	5263.15	1.13	STAD
TTF2|8458	15.78	4.15	NaN	782.16	820.89	397.39	1.05	STAD
CHST1|8534	15.48	4.08	779.16	NaN	247.22	69.01	1.84	STAD
CBLN4|140689	15.32	4.04	779.21	NaN	12.56	0.40	4.96	STAD
LBH|81606	15.31	4.04	666.20	NaN	2102.57	731.75	1.52	STAD
CILP|8483	15.92	4.18	NaN	NaN	388.78	12.91	4.91	THCA
TMEM90B|79953	14.05	3.75	NaN	NaN	150.64	21.28	2.82	THCA
PALM2|114299	13.25	3.56	NaN	NaN	39.18	11.91	1.72	THCA
KAZALD1|81621	12.26	3.34	NaN	NaN	57.66	23.25	1.31	THCA
CCNI2|645121	12.25	3.33	NaN	NaN	37.93	15.23	1.32	THCA
PTH1R|5745	11.95	3.26	NaN	NaN	34.58	5.29	2.71	THCA
FAM111B|374393	11.90	3.25	NaN	NaN	231.15	52.47	2.14	THCA
AGPAT4|56895	11.56	3.17	NaN	NaN	147.16	45.22	1.70	THCA
RPL31P11|641311	11.34	3.12	NaN	NaN	8.56	2.20	1.96	THCA
NRXN2|9379	11.32	3.12	NaN	NaN	269.38	48.68	2.47	THCA
CATSPER2|117155	15.39	4.06	2963.01	NaN	50.94	22.35	1.19	THYM
SLC16A2|6567	15.07	3.98	NaN	3410.15	428.54	112.37	1.93	THYM
SCAI|286205	14.72	3.90	NaN	3410.10	1172.98	430.18	1.45	THYM

C18orf1|753	14.44	3.84	NaN	3410.07	3197.73	869.08	1.88	THYM
LBR|3930	14.37	3.82	NaN	3410.10	4022.03	1451.88	1.47	THYM
CAMK4|814	14.35	3.82	NaN	3410.07	634.34	111.11	2.51	THYM
PRKCB|5579	14.35	3.82	NaN	3410.07	2692.93	671.87	2.00	THYM
LOC153684|153684	14.28	3.80	NaN	3410.07	102.78	35.58	1.53	THYM
TOX|9760	14.20	3.78	NaN	3410.05	1914.60	461.45	2.05	THYM
LOC100128822|100128822	13.94	3.72	NaN	2963.01	386.41	150.34	1.36	THYM
MGAT4A|11320	18.52	4.77	2929.17	3067.01	1479.71	398.10	1.89	UCEC
CDKL2|8999	18.22	4.71	1264.03	NaN	90.15	7.77	3.54	UCEC
TP53TG3B|729355	16.31	4.27	1264.02	NaN	27.53	0.07	8.56	UCEC
PNMA2|10687	15.86	4.17	1264.02	NaN	154.00	4.76	5.02	UCEC
RTBDN|83546	15.75	4.14	1264.02	NaN	41.11	0.67	5.95	UCEC
C20orf196|149840	15.62	4.11	3067.00	1264.06	167.88	75.64	1.15	UCEC
TPTE2|93492	14.92	3.95	3067.16	NaN	7.09	0.15	5.61	UCEC
TMEM63C|57156	14.64	3.88	1264.03	NaN	146.08	8.42	4.12	UCEC
PPA1|5464	14.37	3.82	NaN	1264.05	6490.96	3112.04	1.06	UCEC
SPIRE1|56907	13.89	3.71	1264.02	NaN	737.30	173.67	2.09	UCEC
ACAA2|10449	11.16	3.08	481.04	911.03	1737.44	825.94	1.07	UCS

Table S2 Forty prognostic genes with strong criteria.

The prognostic genes identified at criteria of P[SV]≤10^{-6} and FC(H/L)≥4. The term "Effective" indicates the relationship between gene expression and survival, "Hazard" means a high expression of the gene corresponds to poor survival, and "Protective" means a high expression of the gene corresponds to good survival. The other settings are the same as in the Table-S1.

Gene Symbol | Entrez gene ID	-lg(P[SV])	T-middle-H (days)	T-middle-L (days)	Mean-H (FPKM)	mean-L (FPKM)	log2(FC(H/L))	Effective	Cancer type
ANKRD56|345079	12.10	NaN	1710.00	268.00	73.80	4.40	Protective	KIRC
ASPM|259266	7.48	1100.00	NaN	470.00	56.70	3.05	Hazard	ACC
ASPM|259266	7.79	1830.00	4110.00	262.00	18.80	3.05	Hazard	LGG
ATP6V1C2|245973	11.40	1960.00	NaN	308.00	39.60	2.19	Hazard	KIRC
C1QTNF6|114904	8.51	1960.00	NaN	761.00	267.00	2.26	Hazard	KIRC
C1orf88|128344	8.27	NaN	1190.00	298.00	13.70	4.44	Protective	ACC
C5orf32|84418	6.83	NaN	1190.00	8730.00	2010.00	2.12	Protective	ACC
C7orf29|113763	6.31	2090.00	NaN	529.00	203.00	3.70	Hazard	KIRC
CDC20|991	8.34	1650.00	4110.00	260.00	27.50	3.42	Hazard	LGG
CDC20|991	6.77	1960.00	NaN	216.00	50.90	3.42	Hazard	KIRC
CDC20|991	6.07	1100.00	2510.00	970.00	136.00	3.42	Hazard	LIHC
CDCA8|55143	9.12	1580.00	4110.00	169.00	22.60	2.65	Hazard	LGG
CDCA8|55143	6.77	333.00	826.00	522.00	124.00	2.65	Hazard	MESO

CDK1|983	6.75	1100.00	NaN	1270.00	188.00	2.75	Hazard	ACC
CDK1|983	7.07	1890.00	3730.00	366.00	50.20	2.75	Hazard	LGG
CDK1|983	6.13	359.00	795.00	799.00	256.00	2.75	Hazard	MESO
CEP55|55165	6.48	1100.00	NaN	494.00	63.90	2.95	Hazard	ACC
CEP55|55165	7.96	1890.00	4410.00	93.50	9.80	2.95	Hazard	LGG
CEP55|55165	6.19	1960.00	NaN	188.00	48.60	2.95	Hazard	KIRC
CEP55|55165	8.84	360.00	823.00	775.00	198.00	2.95	Hazard	MESO
CILP|8483	9.29	1910.00	NaN	103.00	6.73	8.31	Hazard	KIRC
DKK1|22943	6.86	986.00	2080.00	778.00	60.80	5.39	Hazard	HNSC
ERCC6L|54821	8.59	333.00	823.00	98.40	27.10	2.64	Hazard	MESO
FAM159A|348378	6.72	1890.00	3760.00	14.70	2.88	2.97	Hazard	LGG
FAM160A1|729830	7.63	NaN	1910.00	140.00	39.20	2.84	Protective	KIRC
FAM72D|728833	7.52	1110.00	NaN	94.10	22.40	2.07	Hazard	ACC
FAM72D|728833	6.57	2090.00	NaN	32.10	10.40	2.07	Hazard	KIRC
GSTA1|2938	7.06	NaN	1200.00	52300.00	1290.00	5.34	Protective	ACC
GTSE1|51512	6.03	1080.00	NaN	298.00	43.90	2.76	Hazard	ACC
GTSE1|51512	6.07	2080.00	NaN	119.00	32.80	2.76	Hazard	KIRC
HOXD10|3236	7.27	1570.00	4070.00	45.00	0.10	2.94	Hazard	LGG
IRX5|10265	9.29	1630.00	3980.00	39.70	1.36	3.52	Hazard	LGG

ISL2|64843	13.00	1520.00	4110.00	28.50	0.42	8.51	Hazard	LGG
KCNK5|8645	6.10	NaN	2090.00	1110.00	438.00	3.32	Protective	KIRC
KIF14|9928	7.71	1650.00	4110.00	128.00	14.00	2.80	Hazard	LGG
KIF14|9928	9.03	350.00	823.00	254.00	56.90	2.80	Hazard	MESO
KLRA1|10748	7.08	2090.00	NaN	65.40	15.00	2.35	Hazard	KIRC
LOC388387|388387	10.40	4230.00	1630.00	8.52	0.79	5.30	Protective	LGG
MC1R|4157	12.10	1910.00	NaN	188.00	49.90	2.01	Hazard	KIRC
MCM10|55388	6.44	1100.00	NaN	150.00	17.90	3.07	Hazard	ACC
MCM10|55388	8.03	360.00	795.00	178.00	42.20	3.07	Hazard	MESO
MYBL2|4605	7.28	1980.00	NaN	260.00	43.00	3.73	Hazard	KIRC
MYBL2|4605	8.57	333.00	823.00	1570.00	247.00	3.73	Hazard	MESO
MYEOV|26579	6.11	460.00	1320.00	1930.00	312.00	5.35	Hazard	PAAD
NPTX2|4885	6.56	2650.00	NaN	145.00	4.37	2.86	Hazard	KIRP
OSR2|116039	12.10	1870.00	4450.00	28.30	0.41	2.65	Hazard	LGG
OTOF|9381	11.30	1640.00	NaN	9.07	1.42	3.63	Hazard	KIRC
PPFIBP2|8495	6.96	NaN	1170.00	395.00	85.90	2.20	Protective	ACC
PPFIBP2|8495	6.33	NaN	1960.00	1180.00	428.00	2.20	Protective	KIRC
RCAN3|11123	6.21	1890.00	4110.00	30.40	7.27	3.21	Hazard	LGG
SGOL1|151648	8.08	361.00	824.00	110.00	31.10	2.94	Hazard	MESO

SHCBP1|79801	6.88	1800.00	3760.00	144.00	27.20	2.77	Hazard	LGG
SHCBP1|79801	9.38	333.00	826.00	491.00	161.00	2.77	Hazard	MESO
SHOX2|6474	7.03	1890.00	3760.00	91.20	0.47	5.34	Hazard	LGG
SHOX2|6474	9.69	1910.00	NaN	22.80	1.49	5.34	Hazard	KIRC
UHRF1|29128	9.09	350.00	826.00	534.00	140.00	2.94	Hazard	MESO
USP2|9099	6.94	NaN	1170.00	989.00	120.00	3.04	Protective	ACC

Table S3 Twenty-two prognostic genes with diagnostic capacity.

The genes that have both prognostic and diagnostic values, with criterion of P[SV] ≤ 10^{-4}, indicating the survival difference between the high and low expression groups, and AUC ≥ 0.80, indicating the capacity of the diagnosing cancer. The term $\log_2$(FC(C/N)) is the fold change of average expression between the cancer (C) and the control (N). The term “Effective” indicates the relationship between gene expression and survival, “Hazard” means a high expression of the gene corresponds to poor survival, and “Protective” means a high expression of the gene corresponds to good survival.

Gene Symbol | Entrez gene ID	-lg(P[SV])	AUC	log2(FC(H/L))	log2(FC(C/N))	Effective	Cancer types
C1QTNF6|114904	8.51	0.97	1.51	2.39	Hazard	KIRC
C1QTNF6|114904	5.40	0.95	1.80	2.37	Hazard	LUAD
CDC20|991	6.07	0.97	2.83	4.65	Hazard	LIHC
CDCA8|55143	4.56	0.96	2.24	3.48	Hazard	LIHC
CDK1|983	4.58	0.94	2.02	2.58	Hazard	LUAD
CEP55|55165	6.19	0.94	1.95	2.51	Hazard	KIRC
ERCC6L|54821	3.63	0.99	1.94	3.29	Hazard	LUAD
FAM111B|374393	4.22	0.96	1.94	2.82	Hazard	LUAD
FAM111B|374393	3.25	0.87	2.14	2.18	Protective	THCA
FAM83A|84985	3.19	0.98	3.00	6.87	Hazard	LUAD
FAM83A|84985	4.98	0.90	4.01	4.49	Hazard	PAAD
GTSE1|51512	6.07	0.94	1.86	2.55	Hazard	KIRC

GTSE1|51512	4.95	0.97	1.99	3.32	Hazard	LUAD
KIF14|9928	4.35	0.98	2.28	4.05	Hazard	LUAD
MYBL2|4605	7.28	0.95	2.59	2.86	Hazard	KIRC
MYBL2|4605	3.27	0.95	2.85	4.02	Hazard	KIRP
MYEOV|26579	6.11	0.88	2.63	3.07	Hazard	PAAD
PRSS53|339105	11.79	0.94	2.07	2.58	Hazard	KIRC
RAC2|5880	3.82	0.91	1.71	2.16	Hazard	KIRC
RCAN3|11123	4.29	0.99	1.20	2.74	Hazard	CESC
RTBDN|83546	4.14	0.87	5.95	7.24	Hazard	UCEC
SGOL1|151648	3.35	0.97	2.22	3.59	Hazard	LUAD
SHCBP1|79801	4.09	0.94	1.75	2.47	Hazard	LUAD
SPAG5|10615	3.06	0.99	1.93	3.41	Hazard	LUAD
STC2|8614	5.05	0.93	2.34	3.01	Hazard	HNSC
STEAP1|26872	5.05	0.88	2.57	2.89	Hazard	LUAD
UHRF1|29128	4.86	0.95	1.82	2.61	Hazard	KIRC

Table S4

Manually identified possible associations between the prognostic gene and the mutated genes and pathways.

**Prognostic genes**	**Cancers**	**Mutated pathways/ genes**	**Possible association**	**PubMed ID (PMID)**
C1QTNF6	KIRC; LUAD	AKT	C1QTNF6→Akt pathway→ angiogenesis	21508531
CDK1	ACC; OV; LGG; LUAD	KRAS	KRAS/CDK1 synthetic lethality	26881434
		BRCA2	CDK1→BRCA1/2→DNA repair	27385216
CDC20	LIHC; LGG; KIRC; LGG	TP53	TP53 → chromatin remodeling → CDC20 promoter → suppress CDC20 transcription	23151139; 1210799; 19273532
CDCA8	LIHC; LGG; MESO		CDCA8 → CCND1; BCL2; P21; P27→ cell cycle G1 phase	
GTSE1	ACC; KIRC; LUAD	AKT; EMT	GTSE1→ Epithelial-Mesenchymal transition (EMT)→ AKT pathway and enhance metastasis	30414902
		Hippo; pRb-E2F1	YAP/TAZ-TEAD4→ GTSE1→ cell migration and invasion	28978043
		Focal adhesion	GTSE1 is Required for Focal Adhesion Disassembly in an EB1-dependent Manner	23236459
HOXD10	LGG; KIRP	RHOC; AKT/MAPK	HOXD10 → (suppresses) RHOC/AKT/MAPK pathway (in human cholangiocellular carcinoma)	26260613
IRX5	LUAD; PRAD; BRAC;COAD	TP53		18519790
		Cell cycle	IRX5→CCND1	30031117
KCNK5	KIRC		ERα binds at KCNK5's enhancer	21680658
KIF14	LUAD; LGG; MESO	PI3K / AKT		24784001
		TP53; TGF-beta		28525372
MC1R	KIRC	TP53	TP53→POMC→α-MSH→MC1R	31612033
MYBL2	KIRC; KIRP; MESO	TP53	Overexpression of E2F1, MYBL2, and FOXM1 initiates aneuploidy; TP53 mutations co-associate with the over expression of mitotic transcriptional	29847804

			activators	
MYEOV	PAAD	Tight junction	SOS1/Ras/MEK/ERK cascade controls junction formation; Inhibition of MEK leads decreased MYEOV (Fig. 3C, EMBO Rep. 6(1):87-96)	25394671
NPTX2	KIRP	TP53	NPTX2→ TP53-PTEN pathway→AKT →repressed NFkB activity	24078801
		KIAA1109	function in the regulation of epithelial growth and differentiation, and in tumor development	1719019 4
OSR2	LGG	MAPK (BRAF; PTEN)	TGF-beta→Smad3/4/7→ OSR2 promoter activity ;<br>BRAF mutation→Ras/Raf/MAPK→OSR2 methylation	22542937; https://doi.org/10.1007/978-3-319-64550-6_13
PPFIBP2	ACC; KIRC; BLCA	MAPK	PPFIBP2/RET→ERK→MAPK	30043417
RCAN3	CESC; LGG	TP53	RCAN3 Is in RNA binding and calcium-dependent protein serine/threonine phosphatase regulator activity	
SGOL1	LUAD; MESO		SGOL1→ genome instability	24146025
SHCBP1	LGG; LUAD; MESO		SHCBP1→p-ERK1/2 & Cyclin D→G0/G1 progression (in lung cancer)	27129942
UHRF1	KIRC; MESO	TP53	UHRF1' high expression associates TP53 mutation	24486181
		DNA methylation	Overexpression of UHRF1→ DNMT1 ubiquitination and degradation→Low DNA methylation level→ genome instability	24486181
USP2	KIRC; MESO	EGFR	USP2a (variant)→ enhance EGFR expression	22710717

Figure Legends

Figure S1 Cancers exhibit a great difference in the number of the genes that are associated with survival difference P[SV] and have a diagnostic value (AUC).

A. Gene numbers at different cutoffs of both survival difference and differential expression between cancer and control. For each gene in each cancer, P[SV] was calculated with a log-rank test in the high- and low-expression groups that were obtained by dividing the cancer tissues by the median expression of the gene. AUC is the area under the ROC curves in differentiating the cancer and the control with the gene expression.

B. The pathways that are significantly enriched for the genes whose expression changes greatly among cancer tissues (FC(H/L)≥2). The analysis was done with the SPIA tool.

Figure S2 Forty prognostic genes identified by the criteria P[SV]≤10^{-6} and FC[H/L]≥4.

A. The identification flow chart. P[SV] is from the log-rank test in the high- and low-expression groups that were obtained by dividing the cancer tissues by the gene's median expression.

B. Venn diagram showing the number of the overlapping genes in our study (A) and in the literature (B) (Uhlen M. *et al.* 2017, Science 357). The overlapping genes are *DKK1*, *EMP1*, *EPS8*, *FAM83A*, *MET*, *MGAT4A*, *MYEOV*, and *PGK1*.

C. Subcellular location of the forty prognostic genes.

D. An enrichment analysis for the identified 236 and 40 prognostic genes with the DAVID tool for KEGG pathways and GO terms.

E. The survival curves of the *MYEOV* high- and low-expression cancer groups in PAAD using microarray data (GEO ID, GSE21501).

Figure S3 Sample to demonstrate that a prognostic gene is not necessarily suitable for discriminating cancer tissues from normal tissues.

A. The survival curves of the *DKK1* high- and low-expression cancer groups.

B. Boxplot of the expression of *DKK1* in LUAD tissues and normal tissues. LUAD tissues are divided into high- and low-expression groups based on the median expression.

C. Expression of *DKK1* in each LUAD tissue.

D. Average expression of *DKK1* in 21 cancers and controls.

Figure S4 Survival curves and ROC curves to show the capacity of the genes in both prognosis and diagnosis in specific cancers.

A. The survival curves of the high- and low-expression groups. The difference significance P[SV] and the median survival time are indicated.

B. ROC curves of diagnosis.

Figure S5 Characteristics of *CDC20* in prognosis and diagnosis in 21 cancers.

A. The boxplots indicate gene expression of *CDC20* in normal tissues, and the high- and low-cancer groups.

B. Plot of the prognostic and diagnostic values of *CDC20* in cancers.

Figure S6

Flowchart of identifying the statistical association between the 40 prognostic genes and the 200 frequently mutated genes.

Figure S7 Most frequently mutated genes in cancers.

A. Shown are the top 40 mutated genes for all cancer types. The vertical axis represents the mutation rate, namely a rate of total number of mutations occurring in the gene to the number of all patients in all cancer types.

B. An enrichment analysis for the top 200 frequently mutated genes.

C. Survival time of the patients with the mutations in each gene. The survival time is truncated at a survival probability of 60%; each patient that has a mutation in the gene was counted. Shown is a heat map indicating the distribution of the survival time against the mutation counts. Lightness is proportional to the number of genes.

D. Relationship between the expression level and the mutation rate for each gene. The mutation rate was normalized by dividing the rate by gene length [Kbp]. The expression data (FPKM) were normalized into percentile in each sample (patient). For each gene, its expression percentile was plotted to its mutation rate per base pair.

Figure S1

A

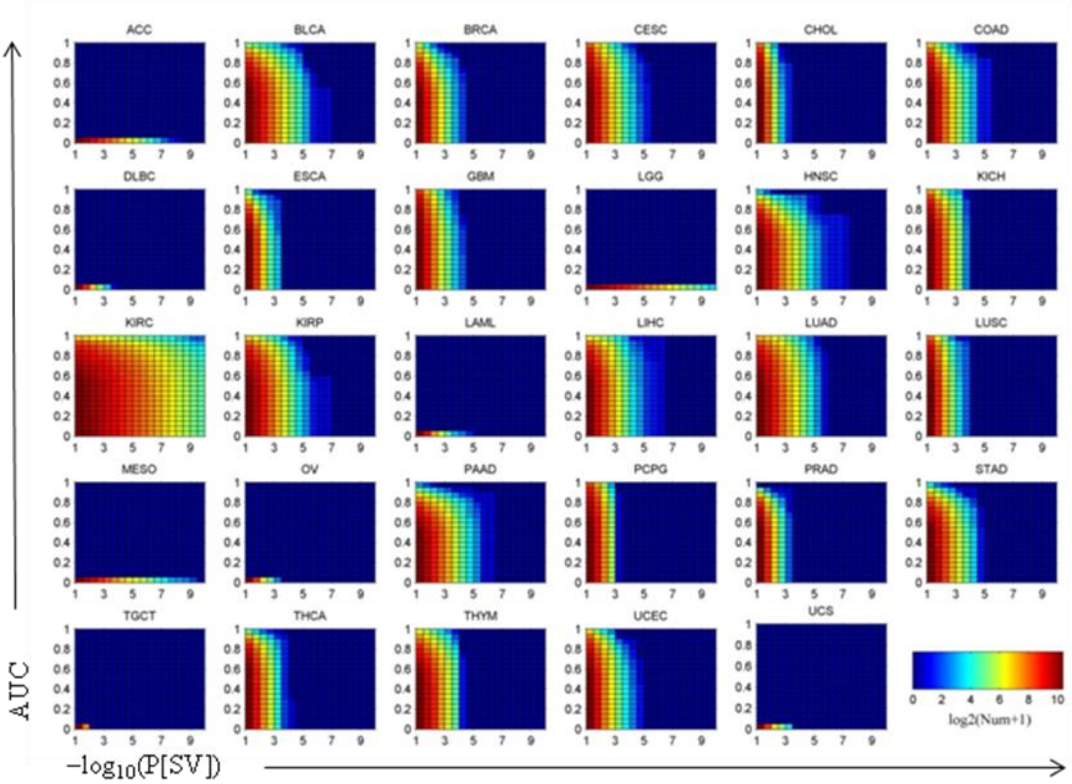

B

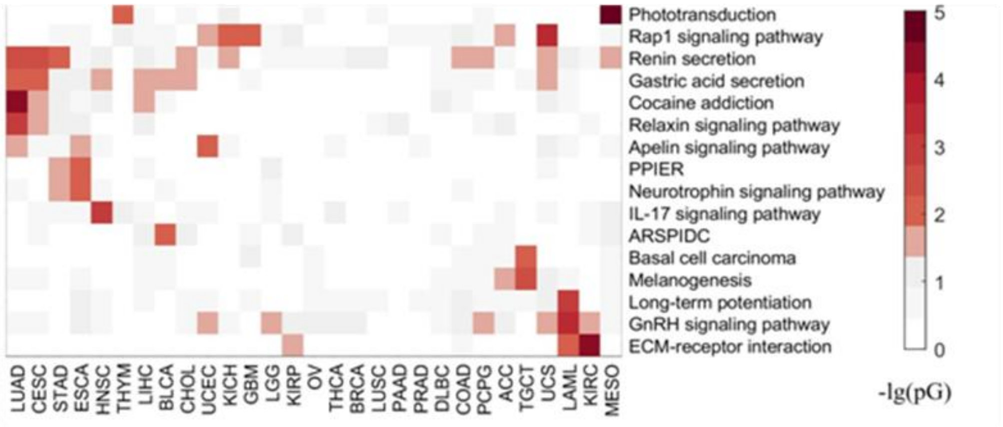

Abbr.:
ARSPIDC, AGE-RAGE signaling pathway in diabetic complications;
PPIER, Protein processing in endoplasmic reticulum

Figure S2

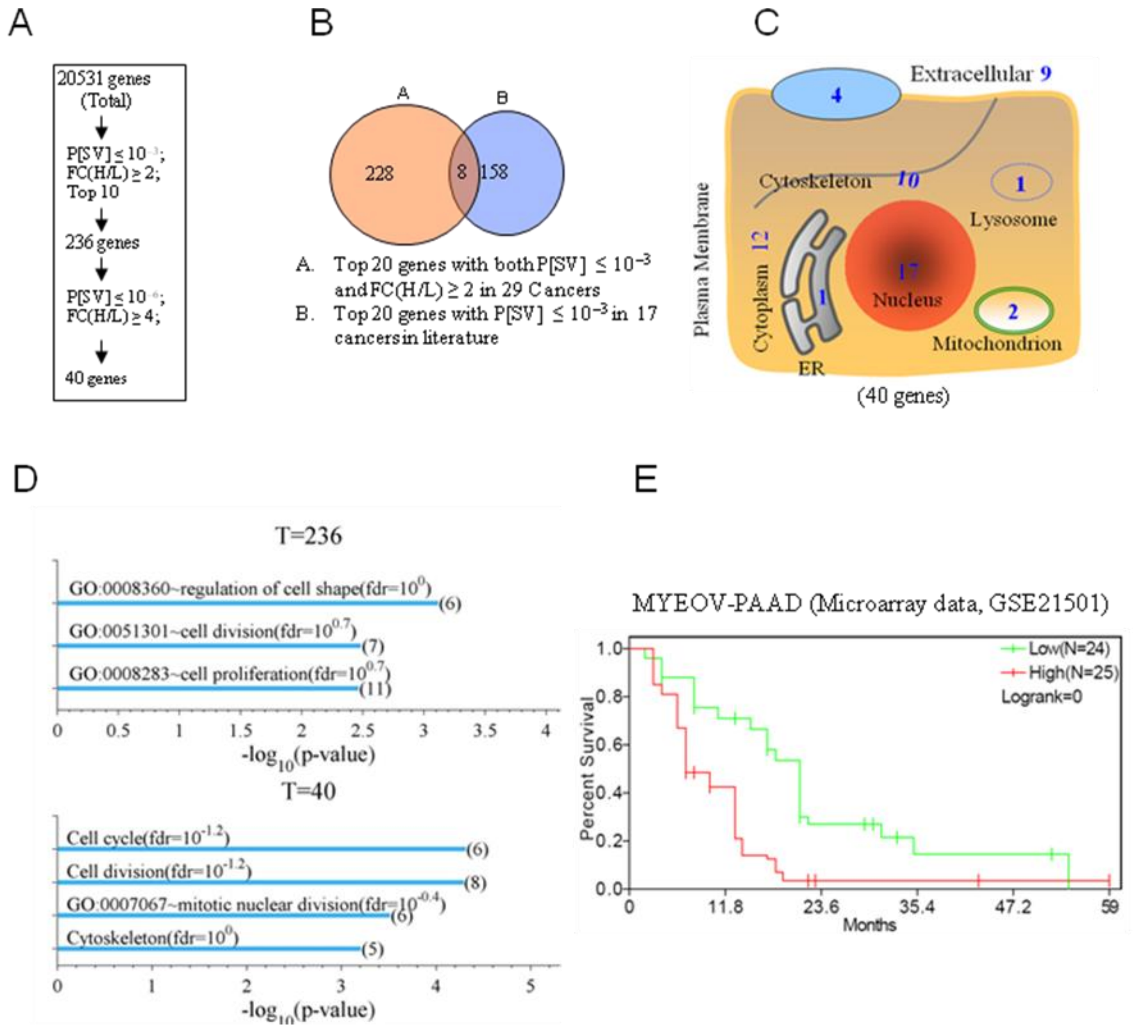
A
20531 genes
(Total)
P[SV] ≤ 10⁻³; FC(H/L) ≥ 2; Top 10
236 genes
P[SV] ≤ 10⁻⁶; FC(H/L) ≥ 4;
40 genes
B
A
B
228
8
158
A. Top 20 genes with both P[SV] ≤ 10⁻³ and FC(H/L) ≥ 2 in 29 Cancers
B. Top 20 genes with P[SV] ≤ 10⁻³ in 17 cancers in literature
C
Extracellular 9
4
Cytoskeleton 10
1
Lysosome
Plasma Membrane
Cytoplasm 12
17
Nucleus
1
2
Mitochondrion
ER
(40 genes)
D
T=236
GO:0008360~regulation of cell shape(fdr=10⁰) (6)
GO:0051301~cell division(fdr=10^0.7) (7)
GO:0008283~cell proliferation(fdr=10^0.7) (11)
-log10(p-value)
T=40
Cell cycle(fdr=10^-1.2) (6)
Cell division(fdr=10^-1.2) (8)
GO:0007067~mitotic nuclear division(fdr=10^-0.4) (6)
Cytoskeleton(fdr=10⁰) (5)
-log10(p-value)
E
MYEOV-PAAD (Microarray data, GSE21501)
Low(N=24)
High(N=25)
Logrank=0
Percent Survival
Months

Figure S3

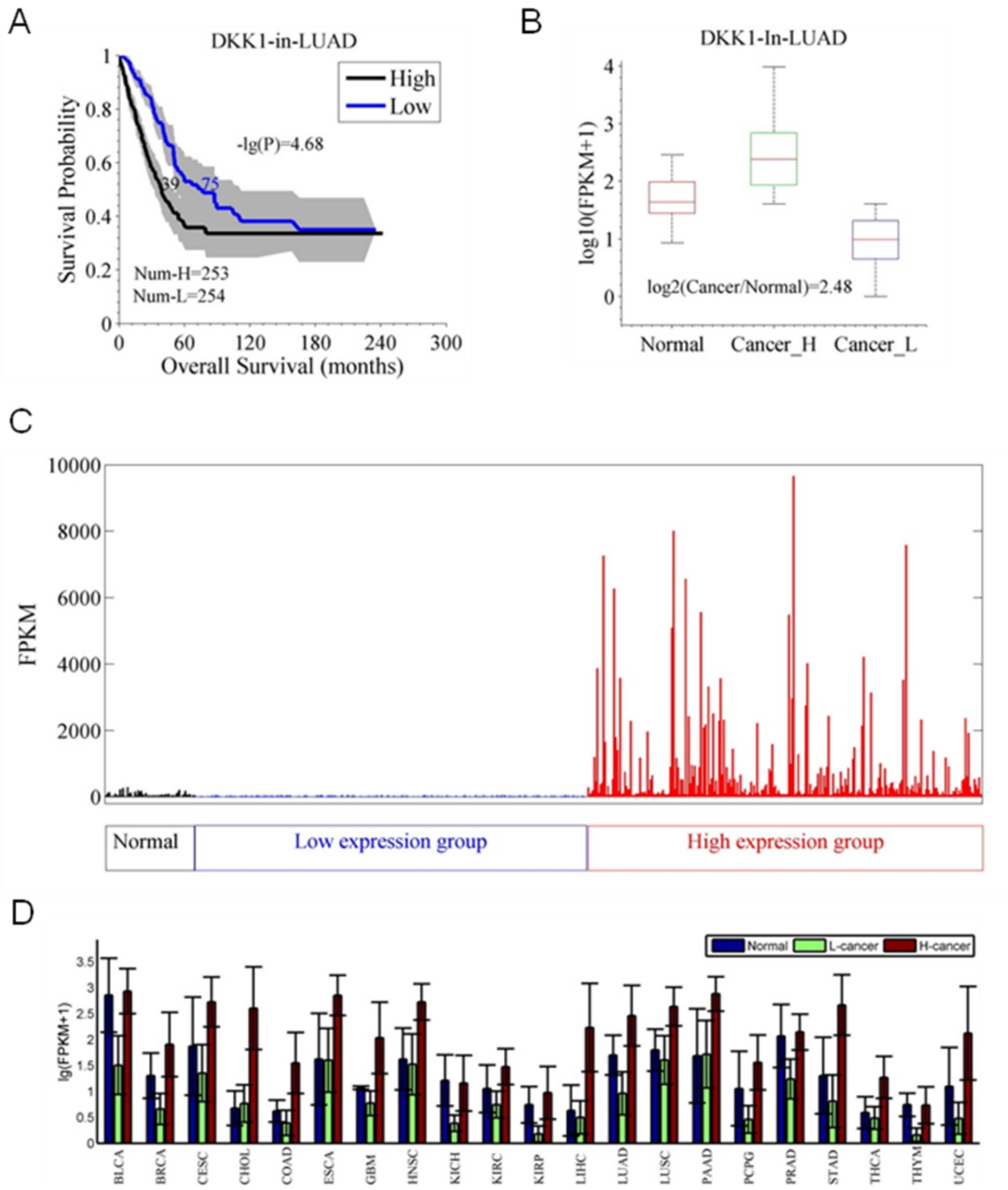
A
DKK1-in-LUAD
High
Low
-lg(P)=4.68
Num-H=253
Num-L=254
Survival Probability
Overall Survival (months)
B
DKK1-In-LUAD
log10(FPKM+1)
log2(Cancer/Normal)=2.48
Normal
Cancer_H
Cancer_L
C
FPKM
Normal
Low expression group
High expression group
D
Normal
L-cancer
H-cancer
lg(FPKM+1)
BLCA
BRCA
CESC
CHOL
COAD
ESCA
GBM
HNSC
KICH
KIRC
KIRP
LIHC
LUAD
LUSC
PAAD
PCPG
PRAD
STAD
THCA
THYM
UCEC

Figure S4

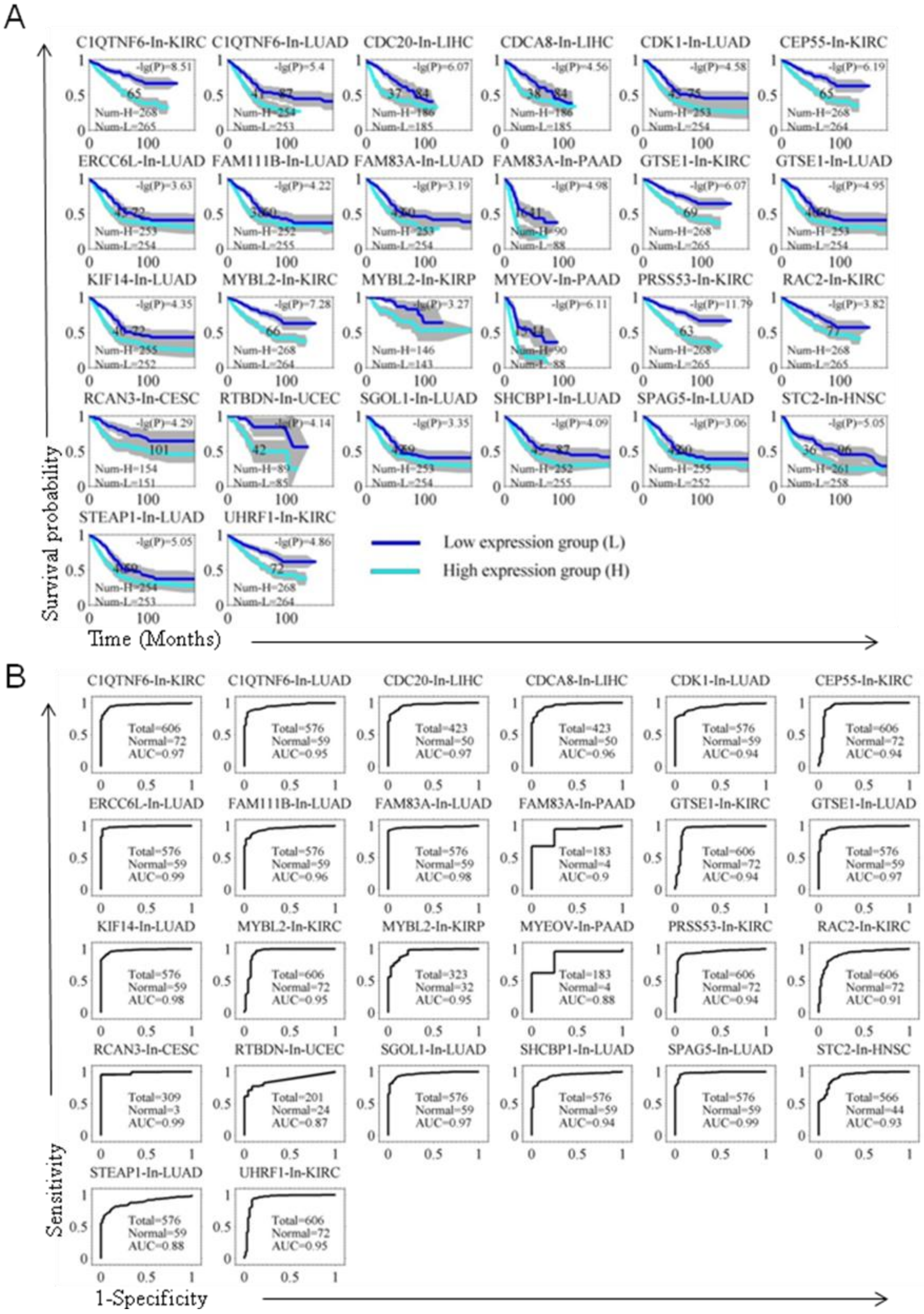
A
Survival probability
Time (Months)
C1QTNF6-In-KIRC
C1QTNF6-In-LUAD
CDC20-In-LIHC
CDCA8-In-LIHC
CDK1-In-LUAD
CEP55-In-KIRC
ERCC6L-In-LUAD
FAM111B-In-LUAD
FAM83A-In-LUAD
FAM83A-In-PAAD
GTSE1-In-KIRC
GTSE1-In-LUAD
KIF14-In-LUAD
MYBL2-In-KIRC
MYBL2-In-KIRP
MYEOV-In-PAAD
PRSS53-In-KIRC
RAC2-In-KIRC
RCAN3-In-CESC
RTBDN-In-UCEC
SGOL1-In-LUAD
SHCBP1-In-LUAD
SPAG5-In-LUAD
STC2-In-HNSC
STEAP1-In-LUAD
UHRF1-In-KIRC
Low expression group (L)
High expression group (H)
B
Sensitivity
1-Specificity
C1QTNF6-In-KIRC Total=606 Normal=72 AUC=0.97
C1QTNF6-In-LUAD Total=576 Normal=59 AUC=0.95
CDC20-In-LIHC Total=423 Normal=50 AUC=0.97
CDCA8-In-LIHC Total=423 Normal=50 AUC=0.96
CDK1-In-LUAD Total=576 Normal=59 AUC=0.94
CEP55-In-KIRC Total=606 Normal=72 AUC=0.94
ERCC6L-In-LUAD Total=576 Normal=59 AUC=0.99
FAM111B-In-LUAD Total=576 Normal=59 AUC=0.96
FAM83A-In-LUAD Total=576 Normal=59 AUC=0.98
FAM83A-In-PAAD Total=183 Normal=4 AUC=0.9
GTSE1-In-KIRC Total=606 Normal=72 AUC=0.94
GTSE1-In-LUAD Total=576 Normal=59 AUC=0.97
KIF14-In-LUAD Total=576 Normal=59 AUC=0.98
MYBL2-In-KIRC Total=606 Normal=72 AUC=0.95
MYBL2-In-KIRP Total=323 Normal=32 AUC=0.95
MYEOV-In-PAAD Total=183 Normal=4 AUC=0.88
PRSS53-In-KIRC Total=606 Normal=72 AUC=0.94
RAC2-In-KIRC Total=606 Normal=72 AUC=0.91
RCAN3-In-CESC Total=309 Normal=3 AUC=0.99
RTBDN-In-UCEC Total=201 Normal=24 AUC=0.87
SGOL1-In-LUAD Total=576 Normal=59 AUC=0.97
SHCBP1-In-LUAD Total=576 Normal=59 AUC=0.94
SPAG5-In-LUAD Total=576 Normal=59 AUC=0.99
STC2-In-HNSC Total=566 Normal=44 AUC=0.93
STEAP1-In-LUAD Total=576 Normal=59 AUC=0.88
UHRF1-In-KIRC Total=606 Normal=72 AUC=0.95

Figure S5

A

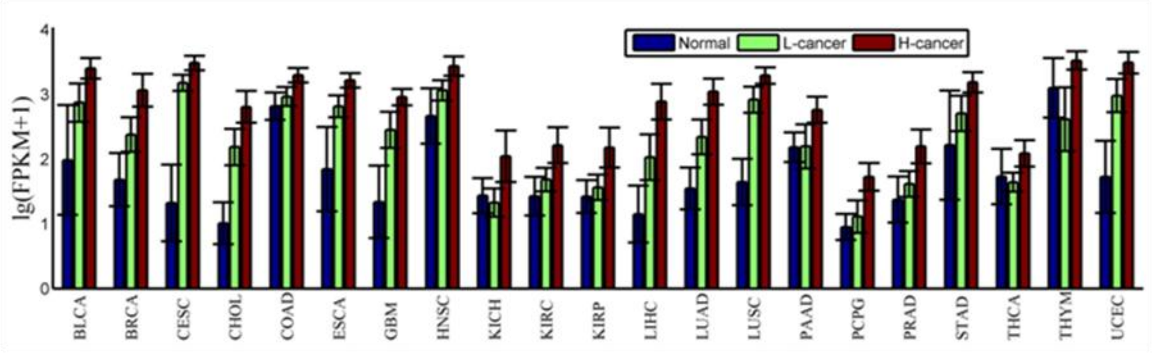
Normal
L-cancer
H-cancer
lg(FPKM+1)
BLCA
BRCA
CESC
CHOL
COAD
ESCA
GBM
HNSC
KICH
KIRC
KIRP
LIHC
LUAD
LUSC
PAAD
PCPG
PRAD
STAD
THCA
THYM
UCEC

B

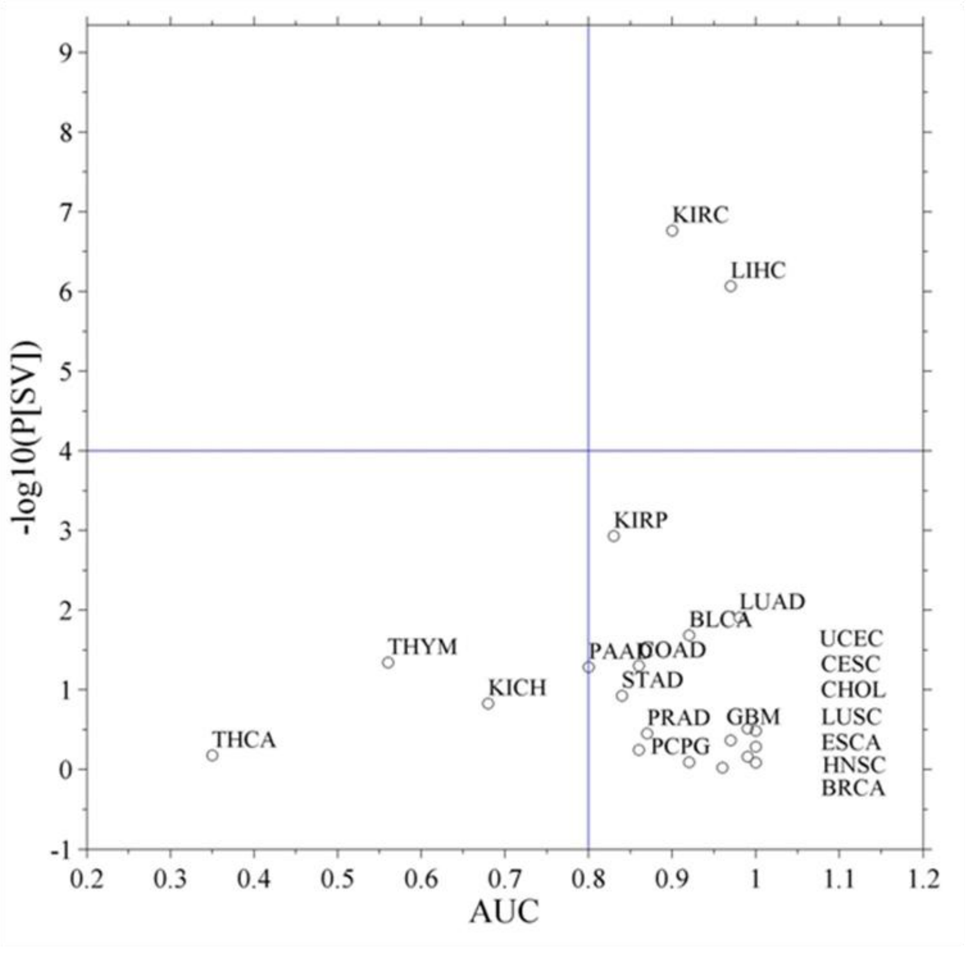
-log10(P[SV])
AUC
KIRC
LIHC
KIRP
LUAD
BLCA
THYM
PAAD
COAD
STAD
KICH
PRAD
GBM
PCPG
THCA
UCEC
CESC
CHOL
LUSC
ESCA
HNSC
BRCA

Figure S6

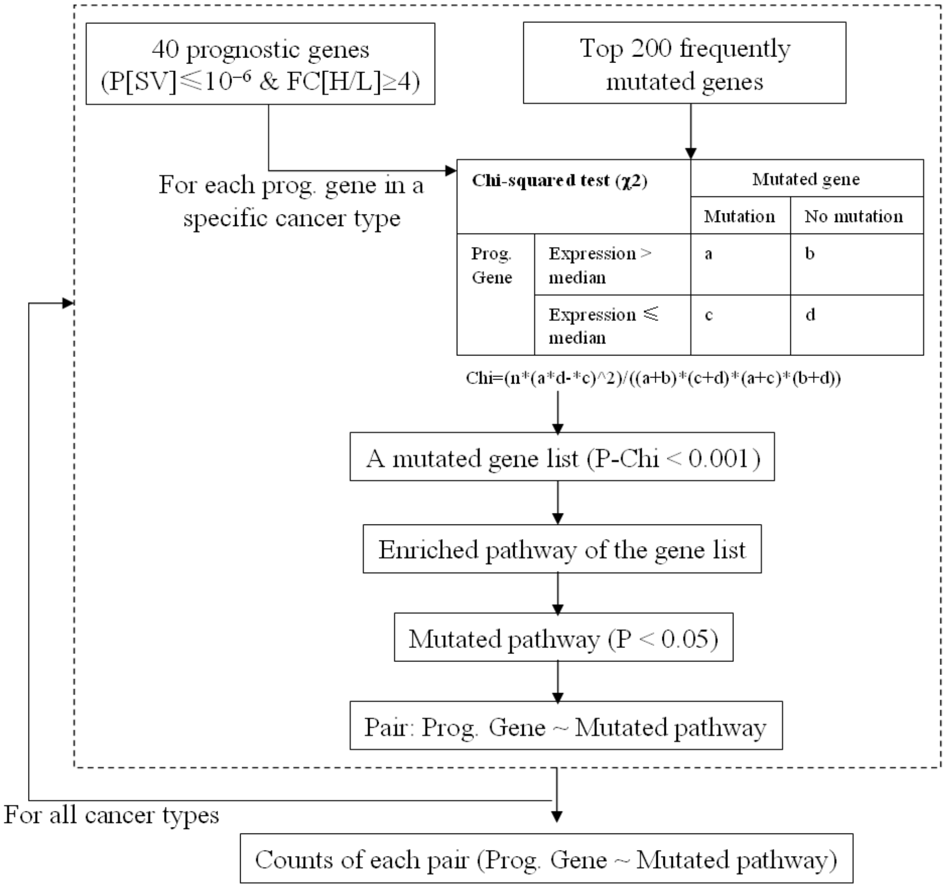
40 prognostic genes
(P[SV]⩽10⁻⁶ & FC[H/L]≥4)
Top 200 frequently mutated genes
For each prog. gene in a specific cancer type
Chi-squared test (χ2)
Mutated gene
Mutation
No mutation
Prog. Gene
Expression > median
Expression ⩽ median
a
b
c
d
Chi=(n*(a*d-*c)^2)/((a+b)*(c+d)*(a+c)*(b+d))
A mutated gene list (P-Chi < 0.001)
Enriched pathway of the gene list
Mutated pathway (P < 0.05)
Pair: Prog. Gene ~ Mutated pathway
For all cancer types
Counts of each pair (Prog. Gene ~ Mutated pathway)

Figure S7

A

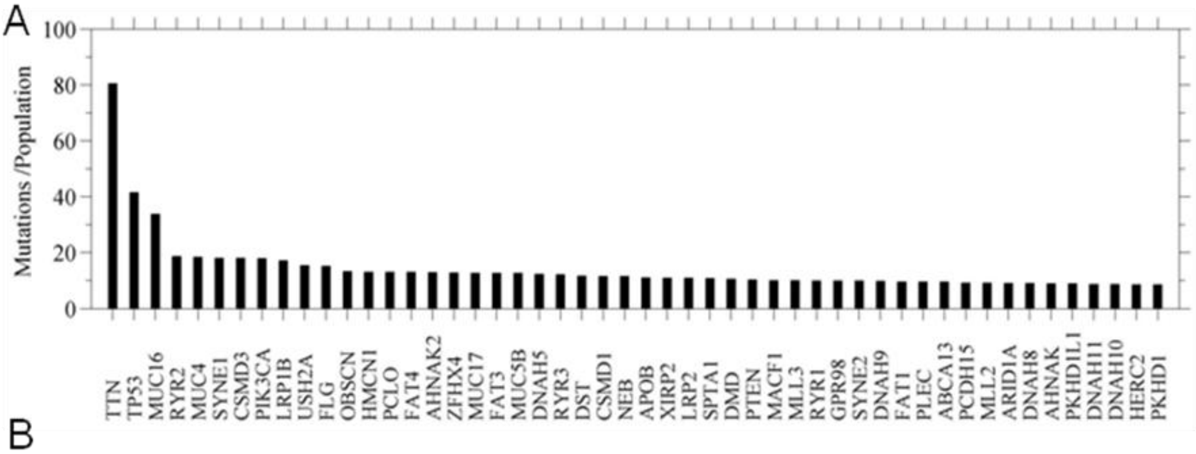

B

The top 200 genes ranked by the maximum mutation rate

Term	Count	PValue	Benjamini	FDR
IPR013783:Immunoglobulin-like fold	24	1.1×10^{-4}	0.0016	0.16
hsa04510:Focal adhesion	12	2.1×10^{-5}	0.0034	0.026
hsa05213:Endometrial cancer	7	2.6×10^{-5}	0.0020	0.032
hsa04512:ECM-receptor interaction	8	5.9×10^{-4}	0.0030	0.071
hsa04151:PI3K-Akt signaling pathway	12	2.0×10^{-3}	0.033	2.30
